# Characterising avenin-like proteins (ALPs) from albumin/globulin fraction of wheat grains by RP-HPLC, SDS-PAGE, and MS/MS peptides sequencing

**DOI:** 10.1186/s12870-020-2259-z

**Published:** 2020-01-29

**Authors:** Yujuan Zhang, Xin Hu, Angela Juhasz, Shahidul Islam, Zitong Yu, Yun Zhao, Gang Li, Wenli Ding, Wujun Ma

**Affiliations:** 1 Australia-China Joint Centre for Wheat Improvement, Western Australian State Agriculture Biotechnology Centre, School of Veterinary and Life Sciences, Murdoch University, Perth, WA 6150 Australia; 2 The Key Laboratory for Quality Improvement of Agricultural Products of Zhejiang Province, School of Agriculture and Food Science, Zhejiang A&F University, Linan, Zhejiang, 311300 Hangzhou China; 3 School of Agriculture, Food and Wine, University of Adelaide, Adelaide, 5005 Australia; 4 Nutritional Crop Physiology, Institute of Crop Science, University of Hohenheim, 70599 Stuttgart, Germany

**Keywords:** Avenin-like proteins, 3D modelling, Gene evolution, RP-HPLC, MALDI-TOF, Post translational modifications

## Abstract

**Background:**

Wheat grain avenin-like proteins (ALPs) belong to a recently discovered class of wheat grain storage protein. ALPs in wheat grains not only have beneficial effects on dough quality but also display antifungal activities, which is a novel observation for wheat storage proteins. Previous studies have shown that ALPs are likely present in the albumin/globulin fractions of total protein extract from wheat flour. However, the accumulation characteristics of these ALPs in the mature wheat grain remains unknown.

**Results:**

In the present study, a total of 13 ALPs homologs were isolated and characterized in the albumin/globulin fractions of the wheat protein extract. A combination of multiple techniques including RP-HPLC, SDS-PAGE, MALDI-TOF and peptide sequencing were used for accurate separation and identification of individual ALP homolog. The C-terminal TaALP-by-4AL/7DS, TaALP-by-4AL/7AS/7DS, TaALP-bx/4AL/7AS/7DS, TaALP-ay-7DS, TaALP-ay-4AL, TaALP-ax-4AL, TaALP-ax-7AS, and TaALP-ax-7DS, were separated as individual protein bands from wheat flour for the first time. These unique ALPs peptides were mapped to the latest wheat genome assembly in the IWGSC database. The characteristic defence related proteins present in albumin and globulin fractions, such as protein disulfide-isomerase (PDI), grain softness protein (GSP), alpha-amylase inhibitors (AAIs) and endogenous alpha-amylase/subtilisin inhibitor were also found to co-segregate with these identified ALPs, avenin-3 and α-gliadins. The molecular weight range and the electrophoresis segregation properties of ALPs were characterised in comparison with the proteins containing the tryp_alpha_amyl domain (PF00234) and the gliadin domain (PF13016), which play a role in plant immunity and grain quality. We examined the phylogenetic relationships of the AAIs, GSP, avenin-3, α-gliadins and ALPs, based on the alignment of their functional domains. MALDI-TOF profiling indicated the occurrence of certain post-translations modifications (PTMs) in some ALP subunits.

**Conclusions:**

We reported for the first time the complete profiling of ALPs present in the albumin/globulin fractions of wheat grain protein extracts. We concluded that majority of the ALPs homologs are expressed in wheat grains. We found clear evidence of PTMs in several ALPs peptides. The identification of both gliadin domain (PF13016) and Tryp_alpha_amyl domain (PF00234) in the mature forms of ALPs highlighted the multiple functional properties of ALPs in grain quality and disease resistance.

## Background

Polymorphic prolamins are composed of several groups of structurally related proteins [1]. Most prolamins are known to contain distinctive N- and C-terminals and repetitive central domains [1]. The prolamin superfamily was defined initially on the basis of a shared skeleton of cysteine residues [1–7]. Recently, Juhász et al. [8] established a new reference map for immunostimulatory wheat grain prolamin and non-prolamin proteins based on the new IWGSC bread wheat reference genome sequence, RefSeq v1.0. Among these re-defined seed-borne allergens, the hydrophobic-seed domain-containing proteins show characteristics of antifungal properties, including cortical cell delineating protein [9], glycine-rich protein [10] and proline-rich protein [11]. Egg-cell secreted protein [12] also has a prolamin-like domain. The lipid transfer protein (LTP) and Non-specific LTP [13] have a LTP-2 domain. The 19 KDa Globulin [14, 15], small cysteine-rich proteins [16] belongs to the Domainless Cys-rich proteins. By contrast, *ω*-gliadins and HMW-GSs are Domainless Cys-poor proteins. The *α*-amylase inhibitors (AAIs), *α*-trypsin inhibitors (ATIs) [17, 18], GSPs [19], Puroindolines [20, 21], *α*-gliadins [22] and avenin-like proteins (ALPs) all contain a Tryp-alpha-amyl domain (PF00234). Meanwhile, the Puroindolines, *α*-gliadins, LMW glutenins, *γ*-gliadins and ALPs also have a Gliadin domain (PF13016). So far, the functional property of the gliadin domain was unknown, except for its nutrient’s reservoir activity during seeds germination. Meanwhile, the extraction, quantification and identification of the complete profile of these individual prolamin proteins in wheat posed a challenge, due to the complexity of the wheat flour proteins.

Despite the fact that water and salt soluble proteins from cereal grain were traditional extracted using diluted salt solutions [23–32], new methods were adopted to analyse and identify components from these protein groups. Water- and salt-soluble proteins from wheat flour have been characterized using a range of protein analytical methods, including SDS-PAGE, RP-HPLC, and differential precipitation by NH_4_Ac-MeOH followed by acetone enabled separation of the most abundant albumins from the gliadins [33–36]. Purothionins, GSP, and several AAIs proteins, as well as several CM3-type alpha-trypsin inhibitors (ATIs) and one protein related to the avenins from oats were identified in the albumin/globulin fraction [35]. Albumins are known to have many different functions and share different types, e.g. glycoprotein, amylase inhibitors, serpins, purothionins, enzymes such as carbohydrases like *α*- and *β*-amylases, or proteolytic enzymes [37]. In the fraction of albumins, the representatives of individual protein components are shown to have functions in pathogen resistance. Albumins such as AAIs and ATIs [38, 39], serpins [40] and purothionins [41] are considered to have a function of nutrient storage and inhibitors of insect and pathogen attack on the germinating seed. Even though smaller amounts of ALPs were first found in the gluten extracts in other studies [42, 43], the close evolutionary relations of ALPs with AAIs and ATIs and avenin-3, as well as the lack of repetitive motifs compared with other gliadins, suggests that they might also be enriched in the albumin and globulin extracts. However, it is hard to isolate the ALPs from a mixture of wheat storage proteins in an efficient way, and the global ALPs protein accumulation profile in different wheat varieties still remains unknown. Till now, the separation of various subunits of the homeologous ALPs in different wheat varieties were rarely reported.

Gluten are storage proteins found in the starchy endosperm of wheat, barley and rye. In wheat, the ALPs can be detected in the gluten-enriched fraction, including among others a range of gliadins, glutenins, protease inhibitors and LTPs [44]. The ALPs were named due to sequence homology with avenins of oats [45], most closely to avenin-3 [46], and *α*-, *γ*-gliadins [47]. Kasarda et al. [48] characterized a novel ALP called farinin, composed by two disulphide-linked small polypeptides subsequent to a proteolytic cleavage of a precursor polypeptide at an Asn-Glu (N-E) peptide bond. Researches were primarily on dough functional quality improvement via incorporation of farinins (ALPs) into the glutenin macropolymers (GMPs) [43, 48, 49]. Further, the functional allelic variations of TaALP-7A were found to be associated with better processing quality [50]. In total, with the previous knowledge, modifying ALPs is a potential way to make better dough for grain industry.

ALP proteins and its function in dough quality have attracted an increasing amount of research attention. Gu et al. [51] found that some storage proteins, such as HMW glutenin, globulins, and ALPs, show upregulated expression under water deficient environment, which might benefit bread making quality. A recent proteomic study indicated that drought stress affects the expression of wheat storage proteins, such as gliadins, glutenins and ALPs as early as 3 days after pollination (DAP), moreover, the misregulated expression is associated with cytoskeleton organization and grain quality proteins in developing seeds [52]. Using Mixolab-dough analysis systems, Wang et al. [53] reported that the starch surface proteins (gliadins, b-type ALPs, LMW-GSs, and partial globulins) in common wheat and waxy wheat displayed different performance to mixing and thermal treatment. Recently, many storage proteins, including HMW-GS, gliadins, globulins, ALPs, triticins, and *ω*-secalins have been identified in wheat endosperm and embryo, which displayed differential accumulation at the protein level between two wheat species that are distinct in grain weight and dough quality, suggesting that ALPs are responsible partly for the quality differences [54]. Based on the study by Altenbach et al. [55], the farinins (ALPs) comprised from 2.6 to 3.1% of the protein in the SDS-extractable polymeric proteins (EPP) and 1.9–2.4% of the protein in the SDS-unextractable polymeric proteins (UPP), and they were influenced by post-anthesis fertilizer. Both type b subunits (bx and by) of ALPs were reported to have non-functional pseudogenes in *Brachypodium distachyon* L accessions, *Triticum dicoccoides*, and *T. aestivum* [47, 56]. Most recently, more novel alleles of *ALP* were found in *Aegilops tauschii* Coss. accessions [57]. Others have studied the multi-functional properties of ALPs despite their effects on dough quality. Gao et al. found [58] a potential protein-protein interaction between a stress-responsive transcription factor, TaERFL1a, and a type a ALP by yeast two hybrid library screening under water deficiency conditions. Meanwhile, Zhang et al. [47] have screened the WEW lines for polymorphisms of ALPs and found the relationships between the ALP gene evolution and environmental parameters. Further, a detailed phylogenetic analysis was performed on the genome-wide *TaALPs* genes and its close relatives to wheat and other monocots species [47], suggesting that ALPs might have the protease inhibition activity like *α*-amylase inhibitors (AAIs), yet the substrates of ALPs can be further identified. Zhang et al. have studied the ALPs and its potential Fusarium head blight resistant functions [59], further illustrated their antifungal properties. Other research suggest that ALP type b are minor storage proteins which are important to protect endosperm starch reserves from degradation [60]. It is reported that, a putative ALP type b that comprises a cereal-type AAIs, as well as serpin-Z1C like defence proteins were increased by elevated CO_2_ [60, 61]. Another novel study, indicated induction of one ALP and one chitinase in winter wheat (varieties. Bologna) grains, not only due to increased CO_2_, but might be linked to the microbial populations [62], as in the case of accumulation of some multifunctional storage globulins, which exhibit antimicrobial activity [63].

In this study, we identified the ALPs in two Australian wheat varieties showing different grain quality, and characterized the ALPs and their electrophoretic mobilities, composition and extraction properties using the separation techniques RP-HPLC and SDS-PAGE. We used the peptide and protein mass identification methods MALDI-TOF and MS/MS to distinguish genome-wide different subunits of ALPs.

## Results

### Allelic variations of ALPs in common wheat varieties

**Fig. 1:**
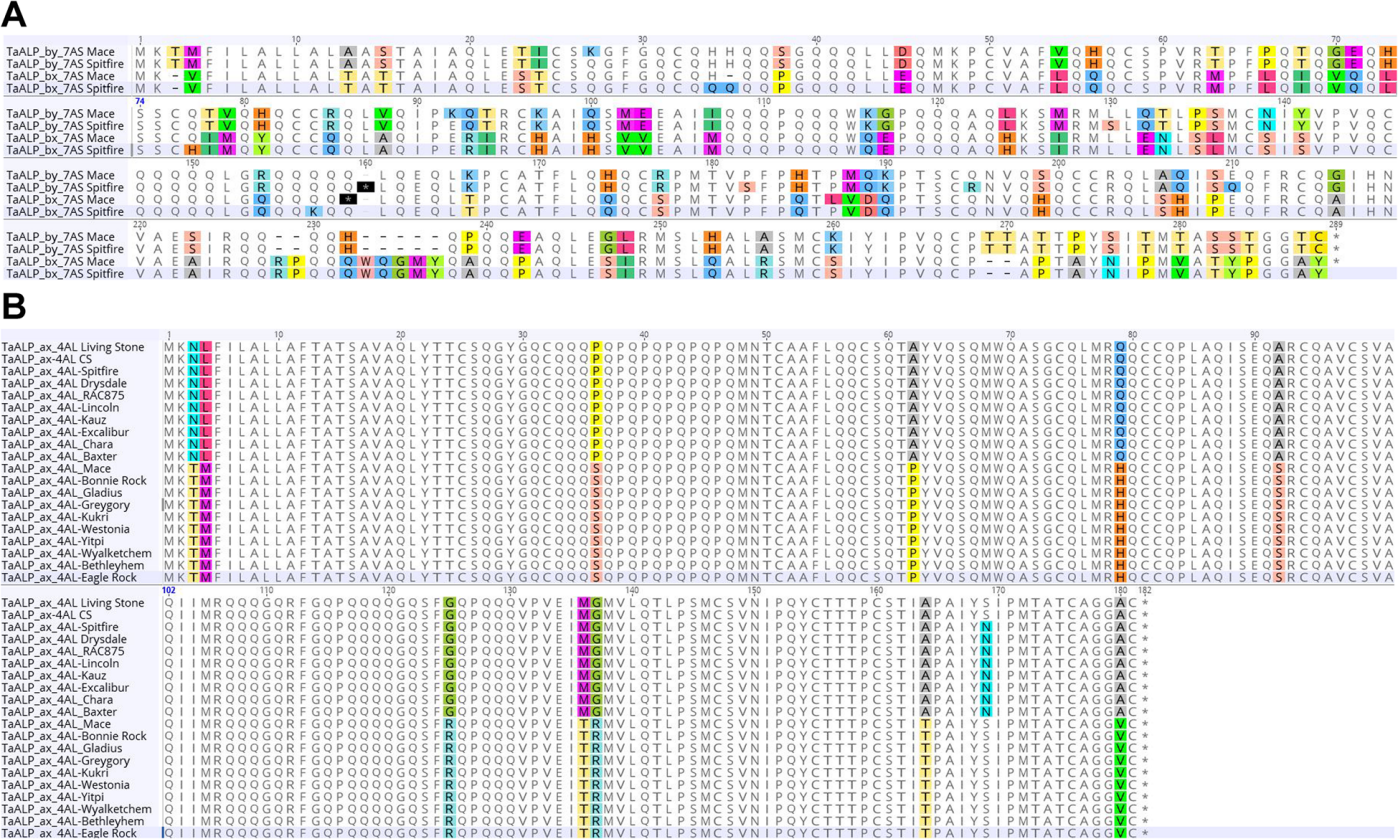
Diversity of *TaALP-bx/by-7AS* and *ax-4AL* genes in common wheat cultivars. **a** Amino acid sequences alignment of TaALP-bx/by 7AS of wheat varieties Spitfire and Mace. **b** Amino acid sequences alignment of *TaALP-ax-4AL* genes in wheat varieties Living Stone, CS, Spitfire, Drysdale, RAC875, Lincoln, Kauz, Excalibur, Chara, Baxter, Mace, Bonnie Rock, Gladius, Greygory, Kukri, Westonia, Yitpi, Wyalketchem, Bethleyhem, Eagle Rock

To identify the allelic variation of *TaALP* genes among different wheat varieties, a total of 15 putative ALP genes were cloned for Sanger sequencing. The allelic variations of the deduced ALPs amino acid sequences in two wheat varieties Spitfire and Mace were revealed by sequence alignment. Amino acid substitutions were identified only for 3 candidate genes: *TaALP-bx-7AS*, *TaALP-by-7AS* and *TaALP-ax-4AL*. As shown in Fig. 1a, Spitfire displayed a pre-mature codon for *TaALP-by-7AS*, while Mace contained a pre-mature stop codon for *TaALP-bx-7AS*. In addition, several non-synonymous mutations between Spitfire and Mace were also observed for both *TaALP-bx-7AS* and *TaALP-by-7AS*. Based on the sequence alignment (Fig. 1b), *TaALP-ax-4AL* alleles can be divided into three types (alleles -a, −b and -c). For this gene, Spitfire and Mace were identified as *TaALP-ax-4AL-b* and *TaALP-ax-4AL-c*, respectively. In addition to the selected three ALP genes, no variation was found between Spitfire and Mace for all other *TaALP* genes present in the wheat genome.

**Fig. 2:**
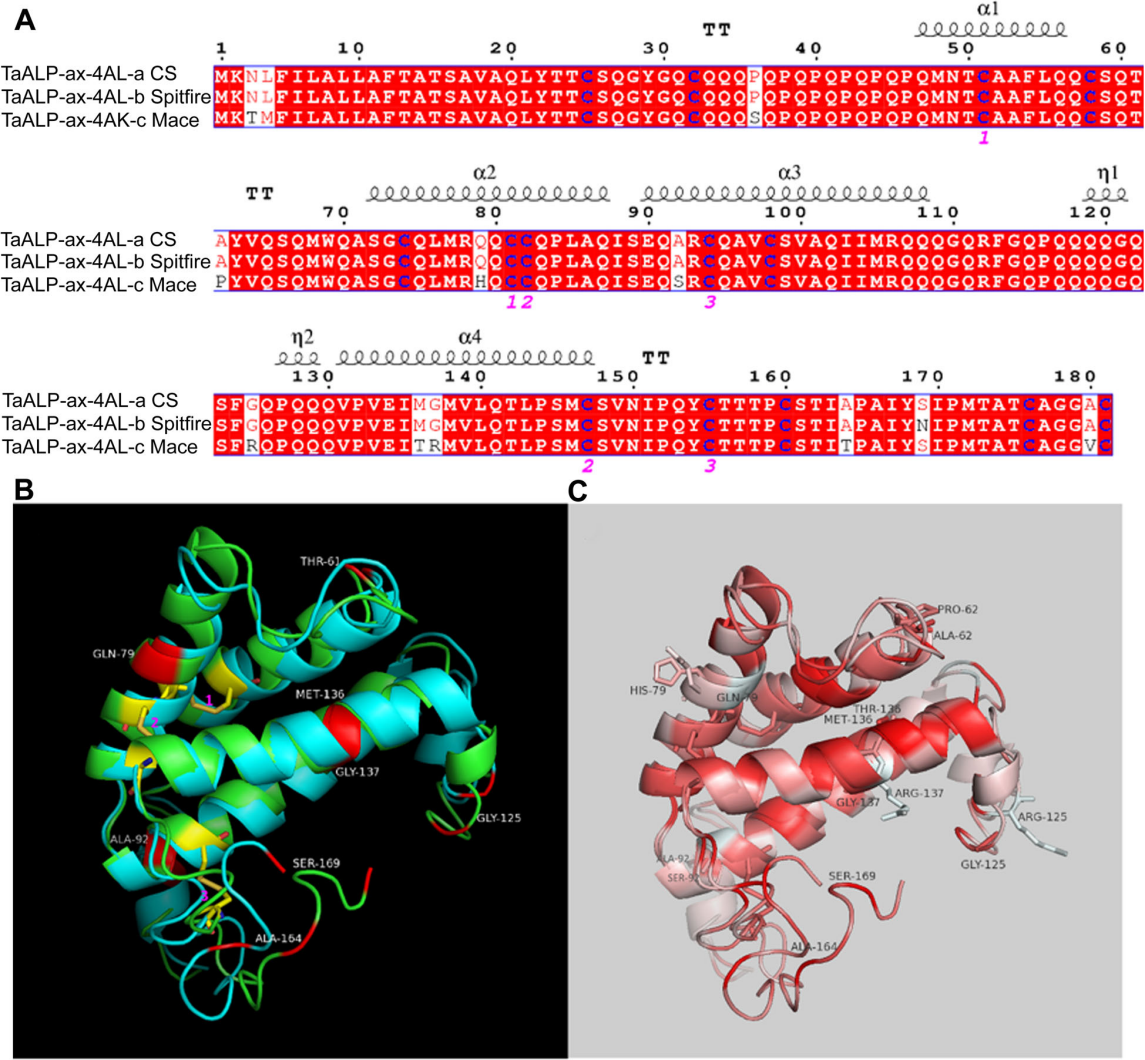
Sequence alignment and protein modelling analyses. **a** Amino acid sequence alignment of TaALP-ax-4AL proteins in CS, Spitefire, and Mace. Cysteine residues were highlighted in blue. Secondary structural elements based on protein modelling were displayed above the sequence alignment. Three predicted disulphate bonds were underlined in pink number 1, 2, and 3. **b** Superimposition of the tertiary structure models of TaALP-ax-4AL in CS (green) and Mace (cyan). Disulphate bonds were displayed in sticks (yellow). The amino acid substitution sites were displayed in red. Only a single substitution (S169 N) exists between CS and Spitfire. **c** Displays the hydrophobicity profile. The substitution site residues with hydrophobicity change were shown in sticks, with red and white colours indicating the most hydrophobic and the most hydrophilic residues, respectively. Protein models were generated using the I-TASSER server [64, 65]. Structure visualization was implemented in PyMol (v1.7.4.5)

The potential protein functional effect of the allelic variations of TaALP-ax-4AL in CS (−a), Spitfire (−b), and Mace (−c) was investigated by sequence alignment and tertiary protein structural modelling analyses. As shown in Fig. 2a, a total of 14 cysteine residues are strictly conserved in the three TaALPs. A total of 11 residue substitutions were identified between CS and Mace. A single amino acid substitution (S169 N) is present in TaALP-ax-4AL proteins from CS and Spitfire. Tertiary structural models were generated for TaALP-ax-4AL in CS and Mace. The protein structure of TaALP-ax-4AL in Spitfire is represented by the CS model. Structural superimposition (Fig. 2b) showed that the tertiary structures of TaALP homologs are generally conserved. Both CS and Mace protein models are consisted of 4 major alpha-helixes, plus 2 short helixes. The single amino acid substitution (S169 N) between CS and Spitfire was located at flexible loop region at the C-terminal, indicating little effect on the protein function. Of the 11 substitutions between CS and Mace, 4 (Q79H, A92S, M136 T, and G137R) are located in the helix regions. Hydrophobicity profile comparison (Fig. 2c) showed that all of these 4 substitutions have caused hydrophobicity changes between the 2 proteins, indicating a potential variance in the enzyme function. The other amino acid substitutions are mainly found in the flexible loop regions, with significant hydrophobicity changes identified in G125R (Fig. 2c). The single amino acid substitution S169 N between TaALP-ax-4AL proteins in CS and Spitfire displays no hydrophobicity change, suggesting potentially identical enzyme function for these 2 proteins.

### Separation and identification of albumin and globulins proteins

**Fig. 3:**
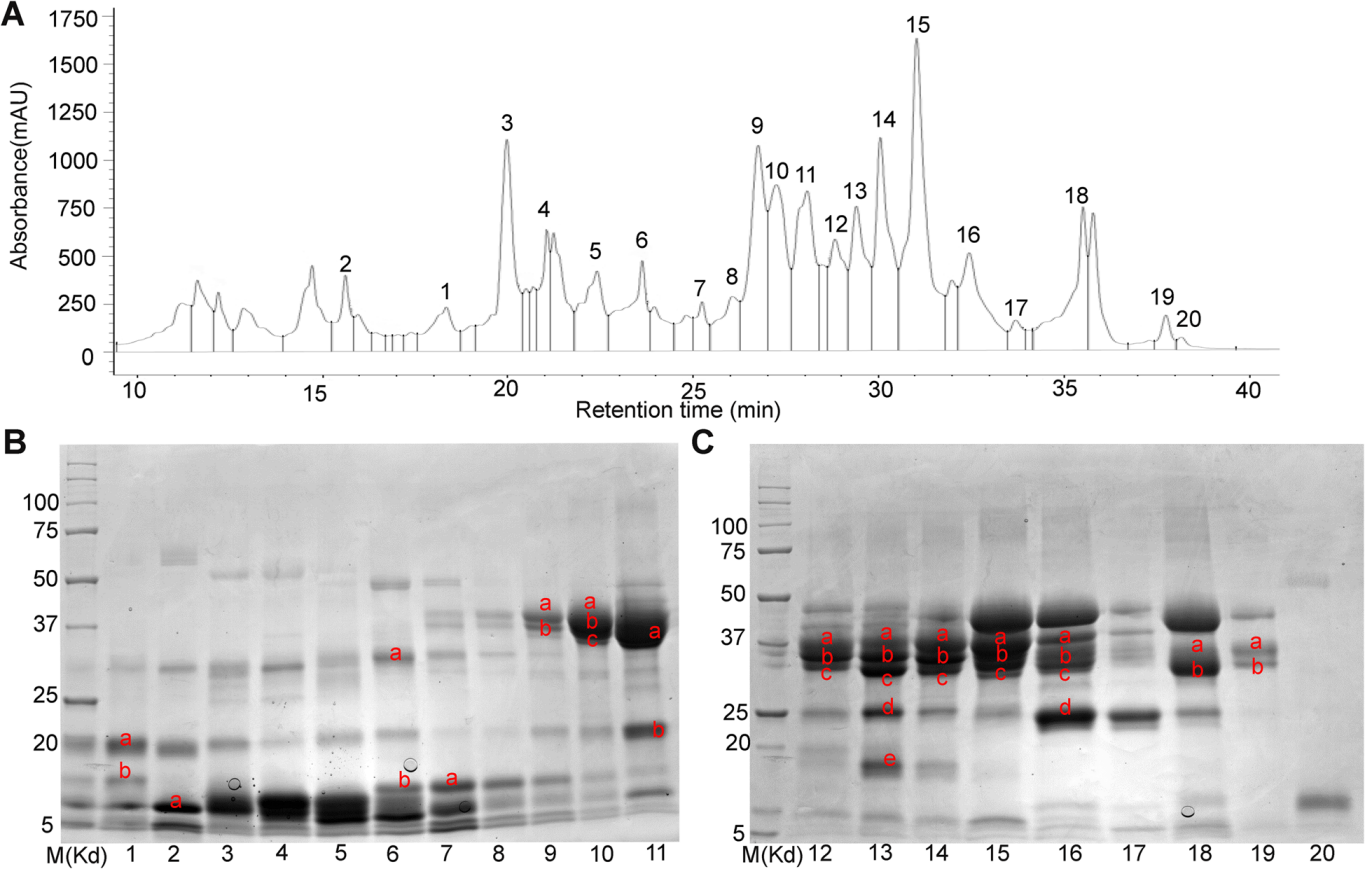
Separation of the wheat flour albumin and globulin extracts. **a** RP-HPLC analyses of albumin and globulin proteins in wheat variety Mace. **b** SDS-PAGE gel separation of albumin and globulin proteins from RP-HPLC peaks 1-11. **c** SDS-PAGE gel separtion of albumin and globulin proteins from RP-HPLC peaks 12-20. The numbers of the horizontal axis indicate the individual HPLC profile peaks; the band was named as peak number plus the characters labelled within each SDS-PAGE gel lanes and were sent for peptide sequencing. We loaded the eluates from RP-HPLC peak 1 (retention time 18.2 min) in the first well of the SDS-PAGE below, while the eluates from peak 2 (retention time 15.8 min) were loaded in the second well of the SDS-PAGE. The original SDS-PAGE gels can be viewed from supplementary data Additional file 5: Figs. S1-S2

To characterize the specific protein composition of certain wheat grain storage protein groups, the albumin and globulin fraction was extracted from wheat flour (variety Mace). A RP-HPLC method was developed for protein separation. As shown in Fig. 3a, a total of 20 HPLC peaks were chosen and collected for further analysis. The collected peak samples were then loaded on SDS-PAGE gels for separation. The one-dimensional SDS-PAGE patterns of each peak were shown in Fig. 3b (Peaks 1–11) and Fig. 3c (Peaks 12–20). Notably, multiple bands were identified for each HPLC peak. Based on the molecular weight (MW) prediction, putative ALPs proteins were identified as protein bands with MW around 17–19 kDa and 28–32 kDa, which corresponded to bands 1a, 1b, 6a, 11b and 13e (Fig. 3b). These proteins displayed a retention time (RT) of 17–26.5 min (Fig. 3b, Additional file 1). In addition, putative AAIs such as CM2 and CM3 were suggested for protein bands (2a, 6b, and 7a, Fig. 3b) of MW below 17 kDa. These proteins fell into peaks 2, 6 and 7 with RT at 23.8–25 min (Fig. 3a, Additional file 1). Similarly, protein bands 1b and 2a were identified as GSP (MW below 17 kDa), which fell into peaks 1 and 2, with RT at 15.6–18.3 min (Fig. 3a, Additional file 1). *α*-gliadins have MW at around 31–40 kDa and were found in abundant amount in peaks 9–14, corresponding to RT at 22.4–27.5 min, whereas *γ*-gliadins (MW 31–40 kDa) were found abundant in peaks 15–19, RT at 28.2–38 min (Additional file 1). Avenin-3 were found from the protein bands 13 d and 16 d (Fig. 3c) in peaks 13 and 16, RT at 29.4–32.5 min (Additional file 1). Taken together, these results suggested that various types and subunits of homologous ALPs were present and separated together with a mixture of AAIs, GSP, *α*-gliadin, and avenin-3.

### Classification of ALPs and other albumin and globulin proteins

**Fig. 4:**
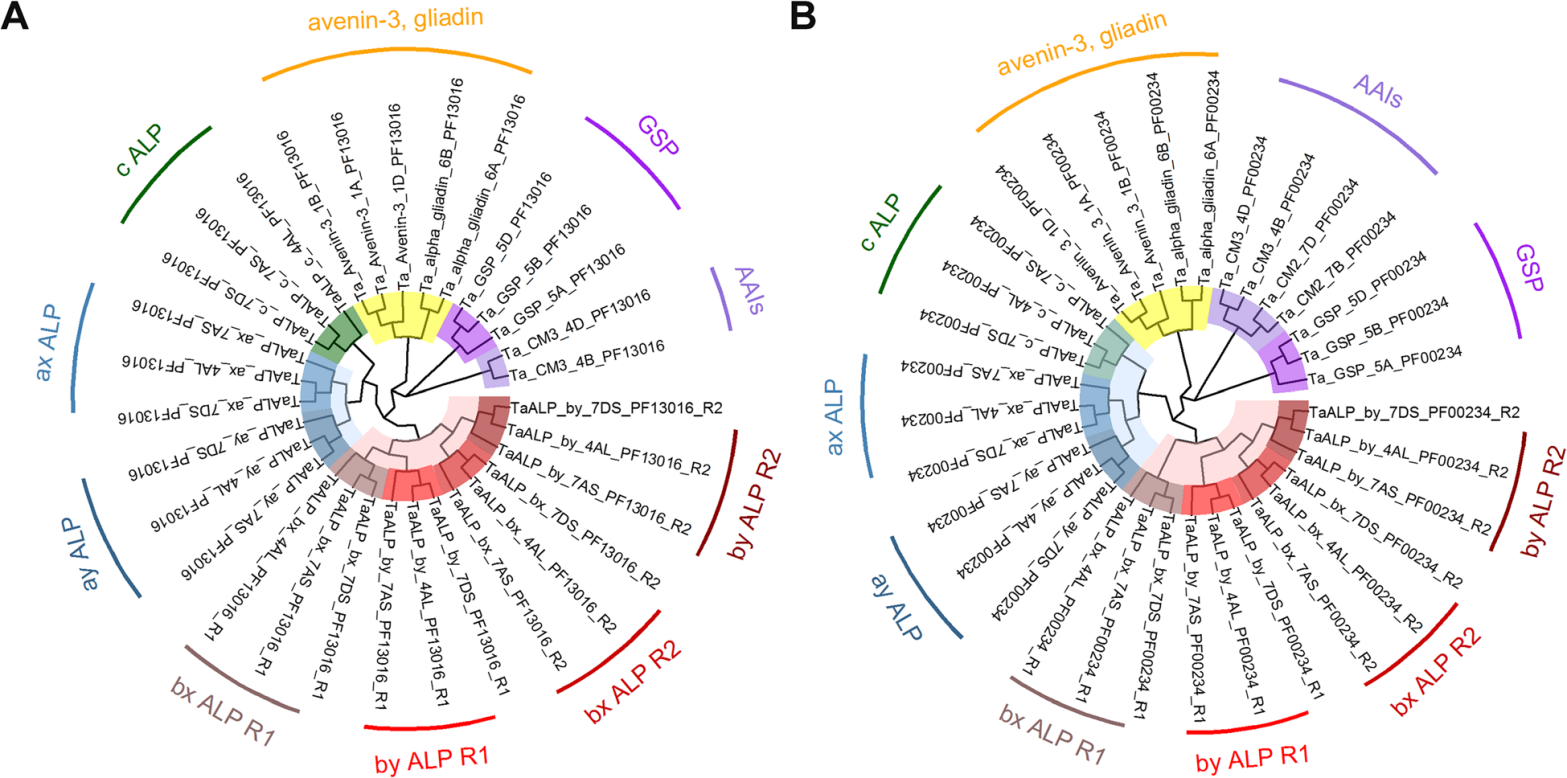
Phylogenetic analyses of the identified protein families from the wheat flour albumin and globulin extracts. **a** Maximum Likelihood (ML) phylogenetic relationship of the bread wheat (*T. aestivum*) PF13016 domain amino acid sequences from ALPs, CM3, GSP, alpha-gliadin and Avenin-3 sequences; **b** ML phylogenetic relationship of the bread wheat (*T. aestivum*) PF00234 domain amino acid sequences from ALPs, AAIs (CM2 and CM3), CM3, GSP, alpha-gliadin and Avenin-3 sequences

ALPs contain a signal peptide and two protein domains: Gliadin domain (PF13016) and Tryp_alpha_amyl domain (PF00234), which are also present in other albumin and globulin proteins, such as the avenin-3, gliadins, GSP and AAIs. To investigate the evolutionary relationship of the different types of ALPs and their relationship with the other co-segregated albumin and globulin proteins, two Maximum likelihood (ML) phylogenies were constructed, based on the sequence alignments of the Gliadin domain (PF13016) (Fig. 4a) and Tryp_alpha_amyl domain (PF00234) (Fig. 4b), respectively. The domain sequences could be found in Additional file 2. Noteworthy, the type b ALP sequences contain 2 cysteine-rich gliadin domains (R1 and R2) [45]. As shown in Fig. 4a and b, the overall topology of both phylogenies were highly consistent and conserved, indicating these two domains had evolved vertically and were present before the divergence among different protein subfamilies. The phylogenetic branches representing GSP, AAIs, avenin-3/gliadin and ALPs could be clearly recognized. In both phylogenies, GSP and AAIs diverged first, followed by the avenin-3 and gliadin. The latter two protein groups displayed a close relationship with each other. In both phylogenetic trees, ALPs were found to be the latest evolved proteins, which further divided into six sub-branches: type c, ax, ay, bx-R1, bx-R2 and by. Of these, type c, ax and ay were grouped together, while the other type b ALPs formed one branch. Interestingly, in both phylogeny cases, the R2 domain of type by ALPs displayed a closer relationship with the R2 domain of type bx ALPs, compared to the R1 domain of type by ALPs. This observation supported that type by ALPs may have originated from the adjoining of type by-R1 and type bx-R2 domains.

### ALP identification by RP-HPLC fractionation in wheat varieties spitfire and Mace

**Fig. 5:**
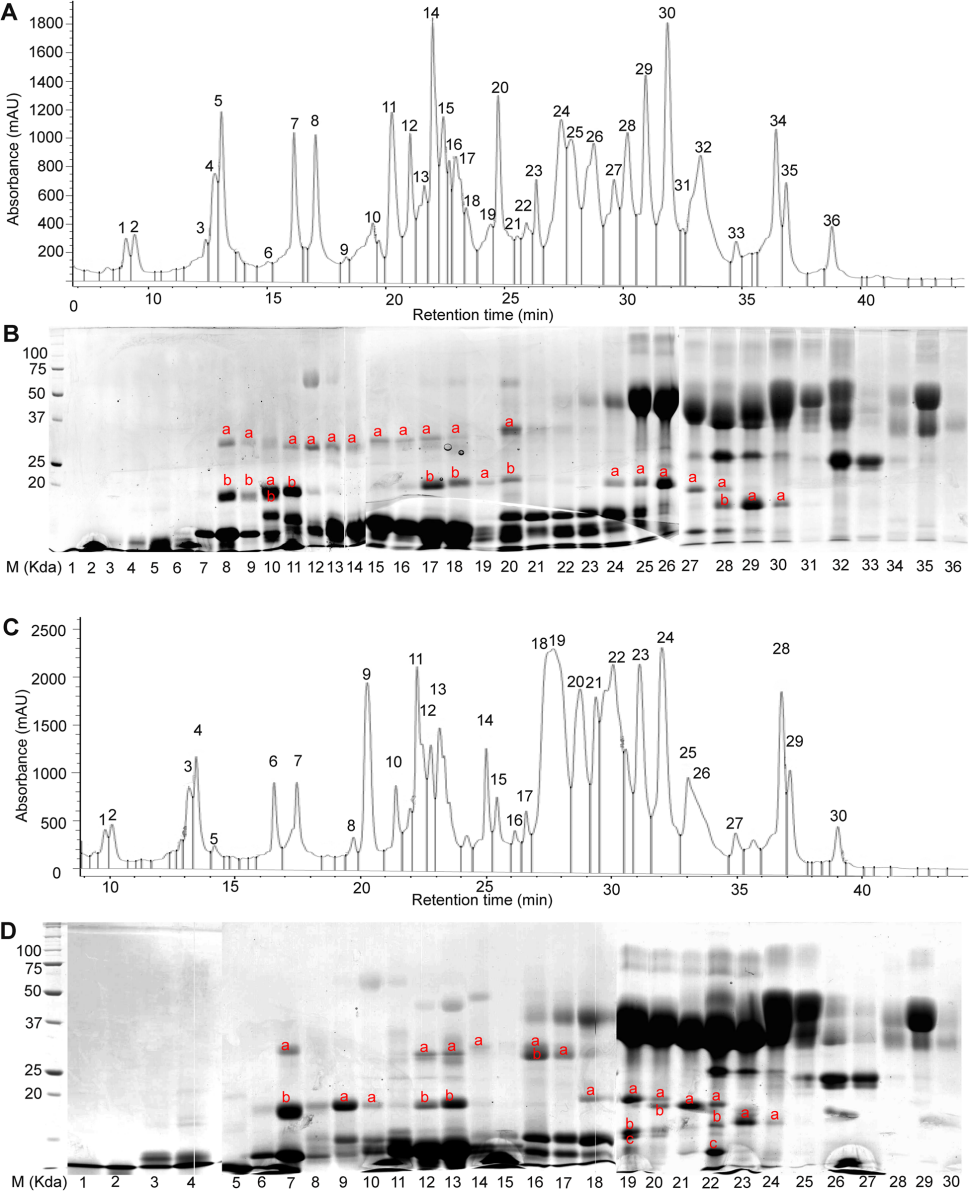
Separation of the wheat varieties Mace and Spitfire flour albumin and globulin extracts. **a** RP-HPLC analyses of albumin/globulin proteins in wheat variety Mace and 36 peaks were collected individually. **b** SDS-PAGE gel separation of the 36 fractions from RP-HPLC. **c** RP-HPLC analyses of albumin/globulin proteins in wheat variety Spitfire and 30 peaks were collected individually. **d** SDS-PAGE gel separation of the 30 fractions from RP-HPLC. The numbers of the horizontal axis indicate the individual HPLC profile fractions; the band was named as peak number plus the characters labelled within each SDS-PAGE gel lanes and were sent for peptide sequencing. The original SDS-PAGE gels can be viewed from supplementary data Additional file 5: Figs. S3-S7

To investigate the variations of ALPs composition in different wheat varieties, total albumin and globulin proteins were extracted from two wheat varieties, Mace and Spitfire. Mace is a variety characterized as high and stable grain yield, whereas Spitfire is featured as slightly lower grain yield but higher grain protein content (≥ 13%) [66]. The two varieties possess different bread-making qualities [66]. For this study, the grain protein content for Mace and Spitfire are 12.14 and 14.22%, respectively. The moisture content for Mace and Spitfire are 12.67 and 12.31%, respectively. Identification of ALPs was carried out using RP-HPLC, SDS-PAGE, peptide-sequencing and MALDI-TOF techniques by following the method described above. The different chromatographic profiles for wheat cultivar Mace (Figs. 3 and 5) were resulted from the use of two RP-HPLC columns (same model). We used the old column for the 20 peaks (from 15 min to 38 min) to target all the albumins and globulins. Later we purchased a new column and obtained 36 peaks (0–38 min) for the same sample. As shown in Fig. 5b, there is no target protein for peaks 1–7 during 0–15 min. So this time discrepancy does not affect our results. Based on this observation, only 15–38 min were targeted in later runs. This second run was used to identify the ALPs specifically.

**Fig. 6:**
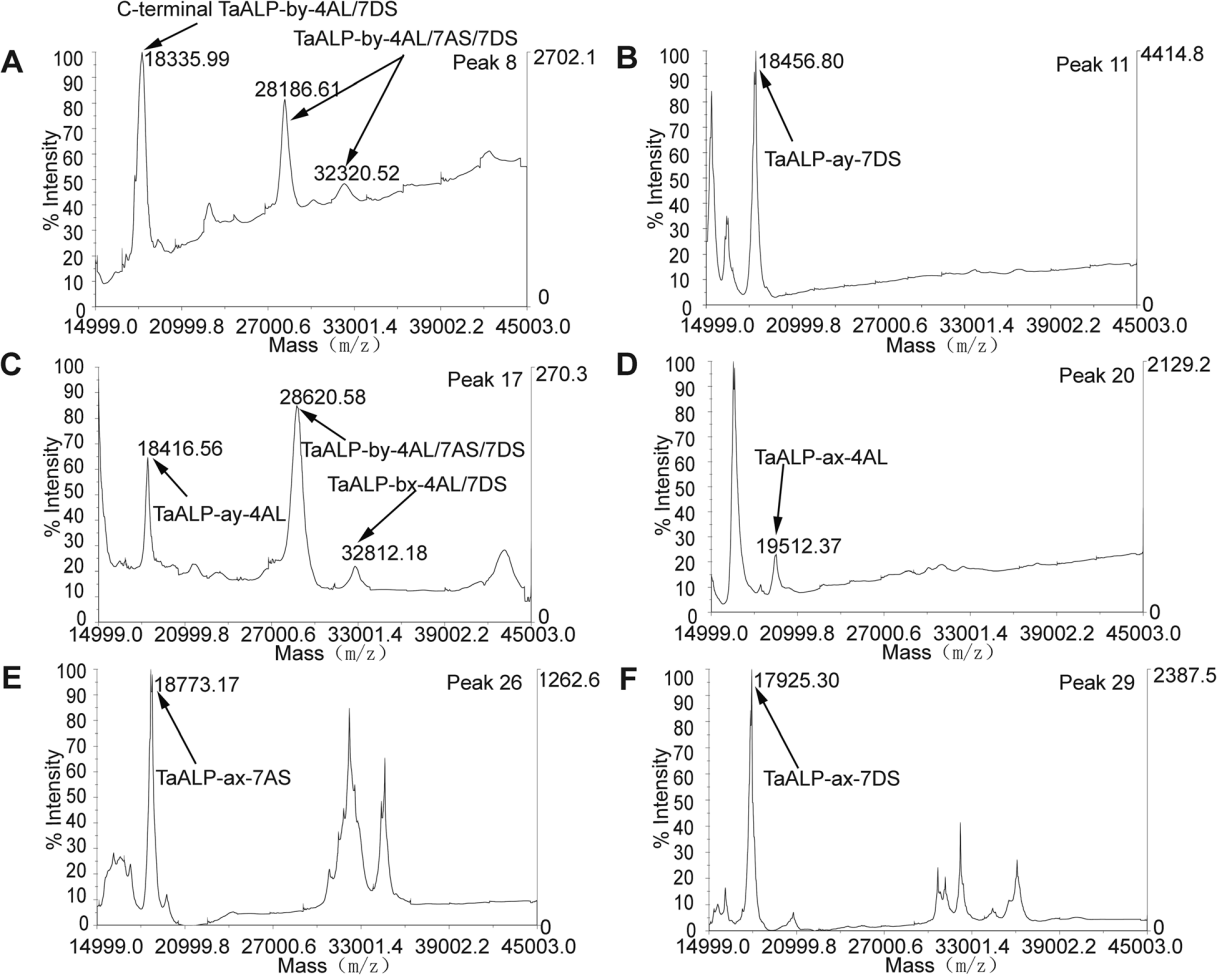
MALDI-TOF profiles of the peaks containing ALP proteins from wheat variety Mace. **a** The MALDI-TOF profile of C-terminal TaALP-by-4AL/7DS and TaALP-by-4AL/7AS/7DS in peak 8. **b** The MALDI-TOF profile of TaALP-ay-7AS in peak 11. **c** The MALDI-TOF profile of TaALP-ay-4AL, TaALP-by-4AL/7AS/7DS and TaALP-bx-4AL/7DS in peak 17. **d** The MALDI-TOF profile of TaALP-ax-4AL in peak 20. **e** The MALDI-TOF profile of TaALP-ax-7AS in peak 26. **f** The MALDI-TOF profile of TaALP-ax-7DS in peak 29. Those peaks not identified as ALP and its derivatives in the MALDI-TOF profile were not labelled

For Mace, a total of 36 elution peaks (Fig. 5a) were identified by RP-HPLC separation. These peak fractions were then loaded on SDS-PAGE gel for further separation. As shown in Fig. 5b, most of the HPLC fractions contained a mixture of proteins with different MWs. Those protein bands with MWs close to or lower than the predicted ALPs MWs (two domains ~ 33 kDa and one domain ~ 19 kDa) were selected as putative ALPs, and were extracted from the PAGE gel for further characterization by peptide sequencing and MALDI-TOF analyses. Peptide sequencing revealed that 27 target protein bands were identified as genuine ALPs (Table 1, Fig. 5a-b, Additional file 1). These protein bands were distributed in 21 HPLC peaks including peak number 8–20 and 24–30. Of these identified ALPs in Mace, both type a and b ALPs were present. Noteworthy, for wheat variety Mace, type a ALPs displayed allelic variations compared to the previously predicted ALP homologues in wheat genome, with *TaALP-ax-4AL-c* allele, while type b ALPs had a pseudogene on chromosome 7A, the *TaALP-bx-7AS* silent allele. In particular, 5 type a ALPs paralogues (TaALP-ay-7DS/4AL, TaALP-ax-4AL/7AS/7DS) were found in 14 bands from HPLC peak number 10–11, 17–20 & 24–30 (Table 1, Fig. 5a-b, Additional file 1). The MWs for these ALPs were verified by MALDI-TOF analyses (Fig. 6b-f), which were consistent with the theoretically predicted MWs for TaALP-ay-7DS (18.42 kDa), TaALP-ay-4AL (18.47 kDa), TaALP-ax-4AL (19.47 kDa), TaALP-ax-7AS (18.75 kDa) and TaALP-ax-7DS (17.90 kDa) (Table 2). In addition, 15 protein bands (8a, 9a, 11a-18a, 20a) were identified as type b (by & bx) ALPs (Table 1). Interestingly, an additional 3 bands (8b, 9b & 10 b) were identified as partial TaALP-by-4AL/7DS, displaying MW at ~ 18.34 kDa (Fig. 6a), whilst typical full length type b ALPs have MW at 31.84 kDa and 31.95 kDa for TaALP-by-4AL and TaALP-by-7DS, respectively (Table 2). This observation indicated the occurrence of inter-domain cleavage specifically for TaALP-by-4AL/7DS. In addition, ALPs with two different MWs (~ 33.32 kDa & ~ 28.19 kDa) were found for protein bands 8a, 9a, 11a and 12a. This observation concerned TaALP-by-4AL/7AS/7DS only, and were verified by MALDI-TOF analyses (Fig. 6a). Intriguingly, a third form of these 3 ALPs (TaALP-by-4AL/7AS/7DS) at MW of ~ 28.62 kDa were also detected during the MALDI-TOF analyses (Fig. 6c). These unusual forms of type-by ALP may have resulted from a cleavage of the full length type b ALP at the myristoylation sites, which were predicted to be present only in some ALPs (Table 3). However, with myristoylation site cleavaging, the theoretically calculated molecular weight for the “by” ALP subunits, TaALP-by-4AL, TaALP-by-7AS, TaALP-by-7DS would be 26.10 kDa, 27.01 kDa and 27.41 kDa, which were smaller than their MALDI-TOF profiles (Table 2). In contrast to the type-by ALPs, the identified type-bx ALPs (TaALP-bx-4AL/7DS), corresponding to bands 13a-18a, and 20a, were all found to be full length ALPs displaying MW at ~ 32.81 kDa (Fig. 6c), whilst full length “bx” ALPs have MW at 32.86 kDa and 32.46 kDa for TaALP-bx-4AL and TaALP-bx-7DS, respectively (Table 2). This observation is consistent with the fact that no myristoylation site has been predicted for type-bx ALPs (Table 3). Even though the myristoylation sites were identified for ALPs, the actual biochemical reactions were hypothetical and need further investigation.

**Table 1:** Identification of TaALPs in wheat varieties Mace and Spitfire

SDS-PAGE bands	HPLC Peak	Retention Time	Sequencing Results
8b	Mace-8	17.035	C-terminal TaALP-by-7DS/4AL
8a	Mace-8	17.035	TaALP-by-7DS/4AL/7AS
9b	Mace-9	18.325	C-terminal TaALP-by-7DS/4AL
9a	Mace-9	18.325	TaALP-by-7DS/4AL/7AS
10a	Mace-10	19.445	C-terminal TaALP-by-7DS/4AL
10b	Mace-10	19.445	TaALP-ay-7DS
11b	Mace-11	20.247	TaALP-ay-7DS
11a	Mace-11	20.247	TaALP-by-7DS/4AL/7AS
12a	Mace-12	21.017	TaALP-by-7DS/4AL/7AS
13a	Mace-13	21.612	TaALP-bx-7DS/4AL
14a	Mace-14	21.967	TaALP-bx-7DS/4AL
15a	Mace-15	22.414	TaALP-bx-7DS/4AL
16a	Mace-16	22.659	TaALP-bx-7DS/4AL
17b	Mace-17	22.941	TaALP-ay-4AL
17a	Mace-17	22.941	TaALP-bx-7DS/4AL
18b	Mace-18	23.375	TaALP-ay-4AL
18a	Mace-18	23.375	TaALP-bx-7DS/4AL
19a	Mace-19	24.414	TaALP-ax-4AL
20b	Mace-20	24.725	TaALP-ax-4AL
20a	Mace-20	24.725	TaALP-bx-7DS/4AL
21a	Mace-21	25.529	TaALP-bx-7DS/4AL
24a	Mace-24	27.387	TaALP-ax-7AS
25a	Mace-25	27.798	TaALP-ax-7AS
26a	Mace-26	28.754	TaALP-ax-7AS
27a	Mace-27	29.614	TaALP-ax-7AS
28b	Mace-28	30.178	TaALP-ax-7DS
28a	Mace-28	30.178	TaALP-ax-7AS
29a	Mace-29	30.914	TaALP-ax-7DS
30a	Mace-30	31.864	TaALP-ax-7DS
7b	Spitfire-7	17.467	C-terminal TaALP-by-7DS/4AL
7a	Spitfire-7	17.467	TaALP-by-7DS/4AL
9a	Spitfire-9	20.266	TaALP-ay-7DS
10a	Spitfire-10	21.406	TaALP-ay-7DS
13b	Spitfire-12	22.796	TaALP-ay-4AL
12a	Spitfire-12	22.796	TaALP-bx-7DS/4AL
13c	Spitfire-13	23.147	TaALP-ay-4AL
13b	Spitfire-13	23.147	TaALP-bx-7DS/4AL
13a	Spitfire-13	23.147	TaALP-bx-7DS/4AL
14a	Spitfire-14	25.004	TaALP-bx-7DS/4AL
16a	Spitfire-16	26.15	TaALP-bx-7DS/4AL
16b	Spitfire-16	26.15	TaALP-bx-7DS/4AL
17a	Spitfire-17	26.601	TaALP-bx-7AS
17a	Spitfire-17	26.601	TaALP-bx-7DS/4AL
17a	Spitfire-17	26.601	TaALP-bx-7DS/4AL
18a	Spitfire-18	27.305	TaALP-ax-4AL
19c	Spitfire-19	27.676	TaALP-ax-7AS peptide 1
19b	Spitfire-19	27.676	N
19a	Spitfire-19	27.676	TaALP-ax-4AL
20b	Spitfire-20	28.743	TaALP-ax-7AS
20a	Spitfire-20	28.743	TaALP-ax-4AL
21a	Spitfire-21	29.378	TaALP-ax-7AS
22c	Spitfire-22	30.058	TaALP-ax-7AS peptide 2
22b	Spitfire-22	30.058	TaALP-ax-7DS
22a	Spitfire-22	30.058	TaALP-ax-7AS
23a	Spitfire-23	31.115	TaALP-ax-7DS
23a	Spitfire-23	31.115	TaALP-ax-7DS
24a	Spitfire-24	32.004	TaALP-ax-7DS
26b	Spitfire-26	33.1	avenin-3-1A
26a	Spitfire-26	33.1	avenin-3-1A

**Table 2:** Summary of the identification of TaALPs in wheat varieties Spitfire and Mace

ALPs	AAs	Cysteine residues	Main Peak of HPLC	Retention time (Min)	MW1 ^a^ (kDa)	MW2 ^b^ (kDa)	MW2 ^c^ (Da)	MW2 ^d^ (Da)
C-terminal TaALP-by-7DS	152	11	Mace-8	17.03	17.39	18.54	–	–
C-terminal TaALP-by-4AL	152	11	Mace-8	17.03	17.44	18.60	–	–
TaALP-ay-7DS	154	14	Mace-11	20.24	16.96	18.42	9133.37	10,070.63
TaALP-ay-4AL	153	14	Mace-17	22.94	17.01	18.47	9198.44	10,135.7
TaALP-ax-4AL	162	14	Mace-20	24.72	18.01	19.47	10,198.54	11,135.8
TaALP-ax-7AS	156	14	Mace-26	28.75	17.29	18.75	9579.93	10,517.19
TaALP-ax-7DS	149	14	Mace-29	30.94	16.44	17.90	9914.25	10,851.51
TaALP-by-7DS	261	19	Mace-8-12	17.03–21.02	29.97	31.95	25,439.88	27,406.12
TaALP-by-4AL	261	19	Mace-8-12	17.03–21.03	29.87	31.84	24,533.1	26,095.2
TaALP-by-7AS	261	19	Mace-8-12	17.03–21.04	29.69	31.67	25,347.9	27,014.14
TaALP-bx-7DS	266	18	Mace-13-23	21.61–26.34	30.59	32.46	–	–
TaALP-bx-4AL	267	19	Mace-13-24	21.61–26.35	30.88	32.86	–	–
C-terminal TaALP-by-7DS	152	11	Spitfire-8	17.46	17.39	18.54	–	–
C-terminal TaALP-by-4AL	152	11	Spitfire-8	17.46	17.44	18.60	–	–
TaALP-ay-7DS	154	14	Spitfire-11	20.26	16.96	18.42	9133.37	10,070.63
TaALP-ay-4AL	153	14	Spitfire-15	23.14	17.01	18.47	9198.44	10,135.7
TaALP-ax-4AL	162	14	Spitfire-21	27.67	17.74	19.20	10,157.53	11,094.79
TaALP-ax-7AS	156	14	Spitfire-23	29.37	17.29	18.75	9579.93	10,517.19
TaALP-ax-7DS	149	14	Spitfire-25	31.11	16.44	17.90	9914.25	10,851.51
TaALP-by-7DS	261	19	Spitfire-8	17.46	29.97	31.95	25,439.88	27,406.12
TaALP-by-4AL	261	19	Spitfire-8	17.46	29.87	31.84	24,533.1	26,095.2
TaALP-bx-7DS	266	18	Spitfire-13-19	21.97–26.60	30.59	32.46	–	–
TaALP-bx-4AL	267	19	Spitfire-13-19	21.97–26.60	30.88	32.86	–	–
TaALP-bx-7AS	266	18	Spitfire-13-19	21.97–26.60	30.52	32.39	24,853.38	26,519.62

^a^Calculated molecular weight of ALPs; ^b^ Calculated molecular weight of ALPs after molecule alkylation; ^c^ Calculated molecular weight of cleaved ALPs; ^d^Calculated molecular weight of cleaved ALPs after molecule alkylation. Note: The theory was that each cysteine residue would combine with one 4-vp molecule and the molecular mass would increase 104.14 Da (the 4-vp molecular mass minus the mass of one hydrogen ion). The cleavage of ALP occurred at the predicted myristoylation cleavage sites

**Table 3:** N-myristoylation site prediction of TaALPs in wheat varieties Mace and Spitfire

ALPs	location	N-myristoylation site
TaALP-ay-4AL	101–106	GQSFGQ
TaALP-ay-7AS	102–107	GQSFSQ
TaALP-ay-7DS	102–107	GQSFGQ
TaALP-ay-7DS	113–118	GQSFGQ
TaALP-ax-4AL	110–115	GQRFGQ
	121–126	GQSFGQ
TaALP-ax-7AS	104–109	GQRFGQ
	115–120	GQSFGQ
TaALP-ax-7DS	108–113	GQSFGQ
TaALP-by-4AL	62–67	GTPFSQ
	239–244	GLRMSL
TaALP-by-7AS_Mace	239–244	GLRMSL
TaALP-by-7DS	239–244	GLRMSL
TaALP-bx-7AS_Spitfire	233–238	GMYQAQ
TaALP-c-4AL	28–33	GSEQCQ
	115–120	GMSQSQ
TaALP-c-7AS	142–147	GIPMAR
	150–155	GGWVCE
TaALP-c-7DS	28–33	GSEQCQ

**Fig. 7:**
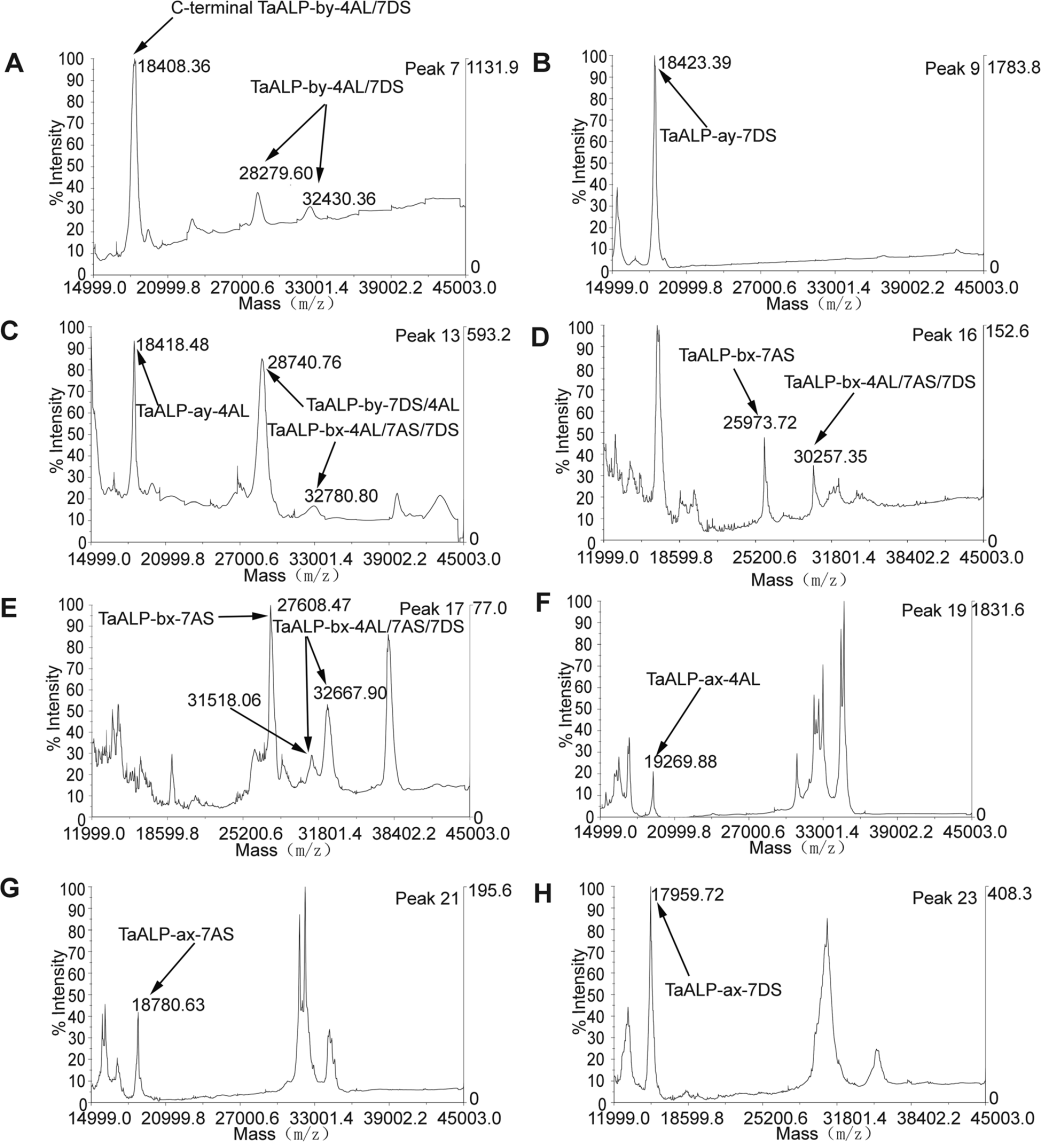
MALDI-TOF profiles of the peaks containing ALP proteins from wheat variety Spitfire. **a** The MALDI-TOF profile of C-terminal TaALP-by-4AL/7DS and TaALP-by-4AL/7DS in peak 7. **b** The MALDI-TOF profile of TaALP-ay-7AS in peak 9. **c** The MALDI-TOF profile of TaALP-ay-4AL, TaALP-by-4AL/7DS and TaALP-bx-4AL/7AS/7DS in peak 13. **d** The MALDI-TOF profile of TaALP-bx-7AS and TaALP-bx-4AL/7AS/7DS in peak 16. **e** The MALDI-TOF profile of TaALP-bx-7AS and TaALP-bx-4AL/7AS/7DS in peak 17. **f** The MALDI-TOF profile of TaALP-ax-4AL in peak 19. **g** The MALDI-TOF profile of TaALP-ax-7AS in peak 21. **h** The MALDI-TOF profile of TaALP-ax-7DS in peak 23. Those peaks not identified as ALP and its derivatives in the MALDI-TOF profile were not labelled

In addition to Mace, similar analyses have been performed on Spitfire. Only 30 HPLC peaks had been identified for the protein extraction in Spitfire (Fig. 5c), which was less than the 36 peaks found for Mace. Further separation by SDS-PAGE gel also showed that most of these HPLC peak fractions contained a mixture of proteins with different MWs (Fig. 5d). Target ALPs proteins were selected using the same strategy, which led to the selection of 24 SDS-PAGE bands for peptide sequencing and MALDI-TOF analyses. Sequencing results showed that ALPs were present in 23 bands distributed in 15 peaks with HPLC peak number 7, 9–10, 12–14, and 16–24 (Table 1, Fig. 5c-d, Additional file 1). Noteworthy, for wheat variety Spitfire, both type b and type a ALPs displayed allelic variations compared to the previously predicted ALP homologues in wheat genome, with *TaALP-bx-7AS-spitfire* functional allele, *TaALP-by-7AS-spitfire* silent allele and *TaALP-ax-4AL-b* allele. Similar with Mace, the same 5 type-a ALPs (TaALP-ay-4AL/7DS, TaALP-ax-4AL/7AS/7DS) were identified in HPLC peak number 9–10, 12–13, 18–24 (Table 1, Fig. 5c-d, Additional file 1), displaying consistent MWs with the computational calculations, which were further confirmed by MALDI-TOF analyses (Fig. 7b-c, f-h). Notably, the calculated MW and the MALDI-TOF profile for TaALP-ax-4AL in Spitfire was 19.20 kDa and 19.27 kDa, respectively, smaller than the homolog Mace allele (Fig. 7f, Table 2). Also, the TaALP-ax-4AL-b Spitfire allele were eluted together with α-gliadins, while the homolog Mace allele TaALP-ax-4AL-c were eluted with AAIs (Fig. 5). In spitfire, partial ax subunits, TaALP-ax-7AS peptide 1 and TaALP-ax-7AS peptide 2 were identified in bands 19c and 22c, respectively, which may have resulted from the presence of myristoylation cleavage sites (Table 3). A total of 8 SDS-PAGE bands (7a-b, 12a, 13a, 14a, 16a-b, 17a) versus 14 bands in Mace were identified as type-b ALPs, which contained both type-by and type-bx (Table 1). The calculated MWs for TaALP-by-4AL and TaALP-by-7DS were 31.84 kDa and 31.95 kDa, respectively (Table 2). However, MALDI-TOF profiles revealed the presence of MWs at 32.43 kDa, 28.28 kDa and 18.41 kDa for these two ALPs (Fig. 7a). This suggested the occurrence of both full length and partial forms for TaALP-by-4AL and TaALP-by-7DS, which has also been observed in Mace. Similarly, the partial forms of these two ALPs may have resulted from the presence of myristoylation cleavage sites (Table 3). In contrast to Mace, in which only the intact form type-bx could be found by MALDI-TOF, both full length and partial forms of type-bx ALPs have been detected in Spitfire. In particular, the calculated molecular weights for TaALP-bx-4AL, TaALP-bx-7AS, and TaALP-bx-7DS were 32.86 kDa, 32.39 kDa and 32.46 kDa (Table 2). Type-bx ALPs with MWs at 32.78 kDa, 32.67 kDa, 31.52 kDa, 30.26 kDa, 25.97 kDa, and 27.61 kDa were identified in the MALDI-TOF profiles (Fig. 7c-e). The detection of MWs at 25.97 kDa, and 27.61 kDa concerned TaALP-bx-7AS only, and supported the occurrence of the myristoylation site cleavage specific for this type-bx ALPs (Table 3), which have not been observed in Mace. Intriguingly, no type-c ALP could be identified for both Mace and Spitfire in the present study.

## Discussion

HPLC, UPLC, and MALDI-TOF technologies have been applied to separate wheat prolamins [67–69]. Procedures for sequentially extracting and recovering protein fractions from small flour samples were previously reported [35]. The NaI-propanol solution solubilized almost all the gliadins, albumins, and globulins, along with traces of glutenin [35]. The present investigation has identified water and salt soluble proteins using multiple techniques including RP-HPLC, SDS-PAGE, MALDI-TOF and peptide sequencing. Apart from the previously characterized proteins in the albumin/globulins extractions, the recent published wheat genome assembly (IWGSC, RefSeq v1.0) have linked the protein peptide sequencing results with more annotated genes. Previously, the identified and characterised proteins included alpha-amylase and protease inhibitors, high molecular weight albumins and other non-storage groups and enzymes, which have specific synthetic, metabolic, regulatory, or protective roles [34, 70]. In this study, we used sequential separation method and identified a range of accumulated ALP subunits in the albumin/globulins fraction. Previously, the identification of ALP type b proteins in the gluten extracts was supported by acquiring the sequences of a reasonable number of tryptic peptides and the matches between measured and expected MW and *pI* [42, 71]. In this study, we identified most ALPs in the albumin/globulin fraction rather than the glutenin and gliadin fractions, which are dominated with typical gluten proteins.

In our analysis, when all the obtained fragmentation patterns are aligned with the respective ALP amino acids sequences, most of the SDS-PAGE bands can be resolved (Additional files 3 and 4). On the contrary, identification of *α−/β-* and *γ*-gliadins and LMW-GS by mass spectrometry tends to give low expectation score, due to the repetitive motifs in the N-terminal regions and proline-rich pattern, which are hard to digest with trypsin [35]. In the case of ALPs identification, the accurate determination of the homologous proteins from 7A, 4A, and 7D is still not achieved due to their highly similar amino acid sequences (> 93%) [47]. Likely, many individual proteins in the region with molecular masses from 33 to 48 kD (mainly gliadins and LMW-GSs) were not resolved by SDS-PAGE, may be due to overlapping of fractions by RP-HPLC peaks (Fig. 3, Additional file 1). Some of the individual ALPs are clearly resolved at apparent molecular masses of 17 to 32 kD and consist of chromosomes 7A/4A/7D loci (Tables 1 and 2, Additional file 1). Protein bands below 16 kD include LMW-albumins, such as members of the complex AAIs and ATIs families that range in mass from 13 to 18 kD. Protein bands of the molecular mass range of 28 to 32 kD include the homologous chromosome 7A/7D/4A-encoded type b ALPs as well as the *α-* and *γ*-gliadins, GSPs, and the LMW-GSs. It is unclear whether the homologous chromosome 4A-encoded TaALP-by-4ALwere resolved in the same bands as TaALP-by-7DS (Figs. 5, 6 and 7). Likely, we could not distinguish C-terminal TaALP-by-4Al from C-terminal TaALP-by-7DS (Figs. 5, 6 and 7).

As a consequence of protein alkylation with 4-vinylpyridine, the theoretical MWs of the ALPs were calculated by adding the alkylation mass of all the cysteine residues in each ALP (each pyridylethyl group increases molecular mass by 105.1 Da). By matching the MWs, distinctive bands of TaALP-ay-7DS and TaALP-ay-4AL were identified, encoded by chromosome 7D and 4A, respectively, both with a molecular mass of approximately 18 kD (Figs. 5, 6 and 7). Likewise, the “ax” ALPs subunits, TaALP-ax-4AL, TaALP-ax-7AS and TaALP-ax-7DS were also clearly identified, with the calculated MWs matching the MALDI-TOF profiles (Figs. 5, 6 and 7). The theoretically calculated MWs of ALPs after cysteine residue alkylation were still different from the MALDI-TOF measured results (Table 2, Figs. 6 and 7). Consequently, the mass difference between them might be correlated to the methionine oxidation, N-terminal acetylation, or phosphorylation that normally occurred during MS analysis [72].

The protein identification indicated that one protein can be identified in several RP-HPLC peaks. The TaALP-ay-7DS proteins were eluted in peaks 5–7; the TaALP-ay-4AL proteins were eluted in peaks 8–10 of wheat variety Mace; the type b ALPs (TaALP-bx-4AL/7DS) were detected in peaks 13–17, 20–21 of wheat variety Mace (Fig. 5). This indicated that overlapping fractions corresponded to almost the entire area of the chromatogram. It is evident that quantification of the individual ALP subunits using the chromatographic profile was not achieved. Furthermore, the co-segregation properties of the different ALPs subunits with other albumin/globulins and gliadins suggests variant physio-chemical properties. ALPs “ay”, “by”, C-terminal “by” and “bx” subunits are more similar to protease inhibitors like AAIs, or the *α*-, *β*- subtilisin-inhibitors and serpins, triticins, while ALPs “ax” subunits are more similar to avenin-3 and *α*-gliadins.

The elution time differences between wheat cultivar Mace and Spitfire might be due to the genotypic differences. For the *TaALP* genes encoding loci, three genes displayed allelic variations and resulted in the different distribution of the corresponding proteins in the RP-HPLC profile and SDS-PAGE gels, as evidenced by the alleles *TaALP-ax-4AL-b* (Spitfire allele) and *TaALP-ax-4AL-c* (Mace allele), with retention times first identified at 24.41 min and 27.30 min, respectively (Table 1, Figs. 5, 6 and 7). This is consistent with the 3D protein modelling results between the two alleles (Fig. 2). The different hydrophobicity profile explains their solubility variances in water and non-polar solvents. The other two alleles are silent alleles identified of *TaALP-bx-7AS-Mace* and *TaALP-by-7AS-Spitfire* encoding gene for Mace and Spitfire, respectively, which resulted in the absence of the actual protein product (Figs. 1, 5, 6 and 7a).

Identification of the PTMs of ALPs was supported by molecular mass based on MALDI-TOF analysis of RP-HPLC fractions. Specifically, the prediction of the myristoylation sites of ALPs (Table 3) supported the post translational cleaving of ALPs at the myristoylation sites. Unfortunately, we have no direct experimental evidence for the myristoylation of the ALPs. Whereas the inter-chain cleavage of “by” ALPs subunits were confirmed by the C-terminal ALP peptides identified on the SDS-PAGE gels, which suggests that ALPs might function as protease interacting substrates. As reported, the C-terminal by-7DS ALP are interacting with *Fusarium graminearum* beta-glucosidase and wheat metacaspase-4 based on a yeast two hybrid assay [59]. Further, the differences between the calculated MWs and the MALDI-TOF analysed results further indicated the occurrence of more than one PTMs, such as the acetylation, formylation, methionine oxidation, phosphorylation, ubiquitination and glycosylation, that are likely to happen to the ALPs (Table 2, Figs. 6 and 7). Future research on the PTMs of ALPs can give more information to this area.

The identities of individual proteins separated by RP-HPLC here were also correlated with those of proteins resolved by others work. Shewry et al. [73] characterized certain seed albumins from different wheat species by N-terminal sequencing and found that several belonged to the AAIs and ATIs family. By using wheat null genetic lines, Singh and Skerritt [33] established the location of several of their genes on individual chromosomes for albumin and globulin proteins. SDS-PAGE analysis of water-soluble proteins indicated the chromosomal location of polypeptides and proteins of different molecular weight were assigned on and 1D, 2A, 2B, 2D, 3AL, 3BS, 3DS, 4AL, 4BS, 4DS, 4DL, 5DL, 6DS, 7BS or 7DL [33]. In our study, besides the identification of ALPs on chromosome arms 7DS, 4AL, and 7AS, it is also displayed in our analysis that other water- and salt-soluble proteins were located to chromosomes 1A/1B/1D (Avenin-3, Gamma-gliadin B, *γ-*gliadins and LMW-GS), 2A/2B/2D (alpha-amylase/subtilisin inhibitor), 3A/3B/3D (Alpha-amylase inhibitor), 4B/4D (AAIs CM3), 5A/5B/5D (GSP), 6A/6B (*α-*gliadins), 7A/7B/7D (60S acidic ribosomal protein, AAIs CM2). Immunological and N-terminal sequencing characterisation identified most of the water-soluble proteins belonged to a family of *AAIs*, serine carboxypeptidase III homologous protein, while the salt-soluble proteins matched with barley embryo globulins, other proteins include, LTP, peroxidase BP-1 precursor and histone H4 proteins [34]. The protein sequences identified could be used for molecular marker development and selection in breeding programmes. Information on the genetics and regulation of this fraction of proteins is necessary to understand their role and function in the grain. It is likely that proteins with similar physio-chemical properties are accumulated in the same fraction. The ALPs identified together with other antifungal proteins in albumin and globulin fraction might indicate similar antifungal functions. This study provided separation solutions for future ALP functional study. The results can be utilized directly by breeding programs aiming for wheat quality and disease resistance improvement.

## Conclusions

With the combination of multiple techniques, we reported for the first time the complete profiling of ALPs present in the albumin and globulin fractions of wheat grain protein extracts. We concluded that majority of the ALPs homologs are expressed in wheat grains. We found clear evidence of PTMs in several ALPs peptides. The identification of both gliadin domain (PF13016) and Tryp_alpha_amyl domain (PF00234) in the mature forms of ALPs highlighted the multiple functional properties of ALPs in grain quality and disease resistance.

## Method

### Plant materials, reagents and chemicals

All wheat materials were provided by Australian Grain Research & Development Corporation. Australian Prime Hard (APH) variety Spitfire and Australian Hard (AH) variety Mace from the 2014–2015 APH field trial were harvested in Macalister of Queensland and Bellata of New South Wales, respectively. The unpolished maturity grain samples were ground whole for protein extraction. All solvents and chemicals used for sample preparation were either HPLC grade or analytical quality, unless stated otherwise. Dithiothreitol (DTT), trifluoracetic acid, 4-vinylpyridine (4VP) and acetonitrile, Sinapinic acid (SA) were purchased from Sigma Chemical Co., St. Louis, MO, USA.

### Gene cloning and sequencing

The primer pairs used in this study were the same as being published by Zhang et al. [47] to amplify *TaALP* fragments from the genomic DNA of wheat varieties, Living Stone, Chinese Spring (CS), Spitfire, Drysdale, RAC875, Lincoln, Kauz, Excalibur, Chara, Baxter, Mace, Bonnie Rock, Gliadius, Greygory, Kukri, Westonia, Yitpi, Wyalketchem, Bethleyhem, and Eagle Rock. PCR amplification cycles consisted of 1 cycle =3 min 95 °C; 35 cycles = 30 s 95 °C, 30 s 60–62 °C, 1 min 72 °C; 1 cycle = 5 min 72 °C. The target PCR products were separated by 1.5% (w/v) agarose gel electrophoresis, and the expected fragments were purified from the gel using a Gel Extraction Kit (Promega, Madison, WI, USA). Subsequently, the purified PCR products were amplified using BigDye@version 3.1 terminator mix (Applied Biosystems) and submitted for Sanger sequencing. Alignment of ALPs was carried out using the MUSCLE add-on tool in Geneious Pro software (v10.2.2).

### Sequence alignment and protein modelling

Amino acid sequence alignment was carried out using the Multiple Sequence Alignment tool [74] at http://multalin.toulouse.inra.fr/ and was further annotated using the ESPript 3.0 tool [75]. Tertiary structure modelling was performed using the template threading method with default parameters implemented at the I-TASSER server [64, 65] at (https://zhanglab.ccmb.med.umich.edu/I-TASSER/). The structure templates identified and used for modelling include PDB: 2LVF, 1W1Q, 1PSY, 1SM7 and 5 U87. Five models were generated for each submitted amino acid sequence, of which, the top-ranking model was used for structural analyses. The selected models for TaALP-ax-4AL in CS and Mace have C-score at − 2.18 and − 2.26, respectively. Considering the flexible and un-modelled N- and C-terminal regions, the overall quality of the generated are of high-quality. Protein structure visualization was implemented using PyMol V1.7.4.5 software [76].

### Phylogeny

The PF00234 and PF13016 domains for AAIs (CM2 and CM3), Avenin-3, alpha-gliadin, GSP, and ALPs were identified by hmmscan search against Pfam database [77] and used for phylogeny development. Codon-based CDS sequence alignments and amino acid sequence alignments were performed using MUSCLE software with default settings. The phylogenetic analysis was done using Maximum likelihood (ML) [78] in MEGA7 [79]. The JTT + G (5 categories) amino acid substitution model was used with 500 times bootstrapping test.

### Near infrared transmission spectroscopy (NIRS) analysis

Wheat cultivar Mace and Spitfire grain samples were used for NIRS analysis without grinding. Three replicates were recorded per sample. Grain protein content (%) and Moisture content (%) were determined by NIRS using the CropScan 3000F Flour and Grain Analyser.

### Protein extraction

The albumin/globulin proteins were extracted from 100 mg of flour according to the procedure of Dupont et al. [35]. Briefly, 100 mg of flour was extracted with 1 mL of 0.3 M NaI, 7.5% 1-propanol (NaI-propanol), and centrifuged at 4500 g for 10 min, after two extractions, the supernatant fractions were pooled in 15 mL tubes, precipitated with four volumes of ice-cold (− 20 °C) NH_4_Ac-MeOH (0.1 M ammonium acetate in 100% methanol), stored at − 20 °C for at least 48 h, and centrifuged as above. The supernatant fluids were transferred into 50 mL tubes and precipitated with four volumes of ice-cold acetone and incubated at − 20 °C overnight. Following incubation, the fluid was centrifuged as above to yield albumin/globulin fraction pellets. The yield estimation of the extract is 10%.

### RP-HPLC

Freeze-dried protein pellets were dissolved in 500 μL 6 M guanidine HCl (with a concentration of 1 mg mL^− 1^) adjusted to pH 8.0 with TRIS, plus 50 mM DTT, and then alkylated with 4VP, prior to HPLC analysis [35]. Albumin and globulin proteins extracted from Spitfire and Mace seeds were analyzed by RP-HPLC. A 1200 Series Quaternary HPLC-System was used, together with a SB-C8 reversed-phase analytical column (5 μm, 4.6 × 250 mm), and a diode array UV-Vis detector (Agilent Technologies, Palo Alto, CA, USA). The column temperature was set at 40 °C. Two mobile solvents were used for linear gradient separation, with solvent A and solvent B consisted of 0.1% TFA (v/v) in ultrapure water (18 MΩ) and 0.1% TFA (v/v) in ACN, respectively. The flow rate was set at 0.6 mL/min. The protein absorbance was detected at 210 nm wavelength. The elution gradient conditions were set as follows: from 0 to 51 min, eluent B was increased from 20 to 60%; from 51 to 53 min, eluent B was increased from 60 to 80% and then maintained at 80% for 5 min for washing the column, then decreased to the starting B concentration in 1 min and maintained for 10 min for the next run. The injection volume was 100 μL. The proteins eluted from individual peaks were collected with reference to the chromatographic profile captured in real time and pooled from three runs. RP-HPLC chromatographic fingerprint profiles showed no variation between runs, thus the elution of each run could be combined to increase the amount of protein in the final sample for later analysis. Samples were immediately frozen at − 80 °C for 24 h and lyophilized. Lyophilized samples were stored at room temperature before MALDI-TOF and SDS-PAGE analyses.

### MALDI-TOF

MALDI-TOF-MS was used to obtain the mass spectra profile of albumin/globulin fractions obtained from individual HPLC peaks (fractions) with and without 4VP alkylation. The albumin/globulin fraction protein extracts were prepared for MALDI-TOF-MS test, whereas the pelleted RP-HPLC eluted protein samples were diluted 20 times for MALDI-TOF-MS test. Each individual RP-HPLC eluates were lyophilized, the freeze-dried eluates were dissolved with 10 μL ultrapure water, 1 μL was used for MALDI-TOF-MS, and the residues were saved for SDS-PAGE running. Sample preparation was carried out according to the dried droplet method [80], using sinapinic acid (SA) as matrix. The matrix solution was prepared by dissolving SA in ACN/H_2_O/MeOH (60:8:32 v/v) at a concentration of 20 mg/mL. All samples, including the RP-HPLC eluates, the raw albumin/globulin extracts and the alkylated albumin/globulins extracts were mixed with SA at the ratio of 1:9 (v/v) individually, and firstly, 1 μL of this protein-SA mixture was deposited onto a 100-sample MALDI probe tip. After drying, another 1 μL of this protein-SA mixture was added, then dried at room temperature. The mass spectra for each sample was recorded on a Voyager DE-PRO TOF mass spectrometer (Applied Biosystems, Foster City, CA, USA) using a positive linear ion mode at an accelerating voltage of 25 kV and a delay time of 700 ns by capturing 1000 spectra of a single laser shot with a mass range of 15,000–45,000 m/z.

### SDS-page

To identify the ALPs from RP-HPLC eluates, SDS-PAGE was used to separate the protein mixtures of each RP-HPLC eluate, and SDS-PAGE bands of interest were cut for protein peptides sequencing. The 12% SDS-PAGE was prepared following Fling and Gregerson’s method [81]. Pelleted samples of HPLC eluates described above were mixed with 10 μL 2 × laemmli sample buffer SDS loading buffer (Bio Rad). Electrophoresis was carried out in a modified Laemmli system [82]. Runs were performed with running buffer of 25 mM Tris-HCL, 192 mM glycine and 0.1% SDS at 120 V for 2 h. The gels were stained in Coomassie Brilliant Blue (CBB) solution (R-250). Protein standards (Bio-Rad) were used to estimate the molecular size of the proteins. The gels were scanned by a gel Proteomic Imaging System “Image lab 5.0” (Bio-Rad).

### Protein identification by MS/MS

Protein bands of interest were manually excised from the SDS-PAGE gels and analysed by Proteomics International Ltd. Pty, Perth, Australia. Protein samples were trypsin digested and the resulting peptides were extracted as previously described [83]. For each protein band sample, 125 nanograms of trypsin were added for digestion. The protein spots identification by MS/MS were as previously described [53].

## Supplementary information

**Additional file 1.** Peptide sequencing results of albumin/globulin extracts proteins.

**Additional file 2.** PF00234 and PF13016 domain sequences of albumin/globulin extracts proteins.

**Additional file 3.** ALP proteins identification from wheat cv. Mace.

**Additional file 4.** ALP proteins identification from wheat cv Spitfire.

**Additional file 5.** Original SDS-PAGE gels for Figs. 3 and 5.

## Data Availability

All data generated or analysed during this study are included in this published article and its supplementary information files.

## Abbreviations

ACN: Acetonitrile
CS: Chinese Spring
DAP: Days after pollination
EPP: Extractable polymeric protein
KDa: Kilodalton
MALDI-TOF: Matrix Assisted Laser Desorption/Ionization - Time of Flight
MW: Molecular Weight
RP-HPLC: Reversed phase-High Performance Liquid Chromatography
TFA: Trifluoroacetic acid
UPP: Unextractable polymeric protein
