# Supplementary figures and images for: Characterising avenin-like proteins (ALPs) from albumin/globulin fraction of wheat grains by RP-HPLC, SDS-PAGE, and MS/MS peptides sequencing
Source: BMC Plant Biol. 2020 Jan 29;20:45. doi: 10.1186/s12870-020-2259-z (PMC6988229; doi:10.1186/s12870-020-2259-z)

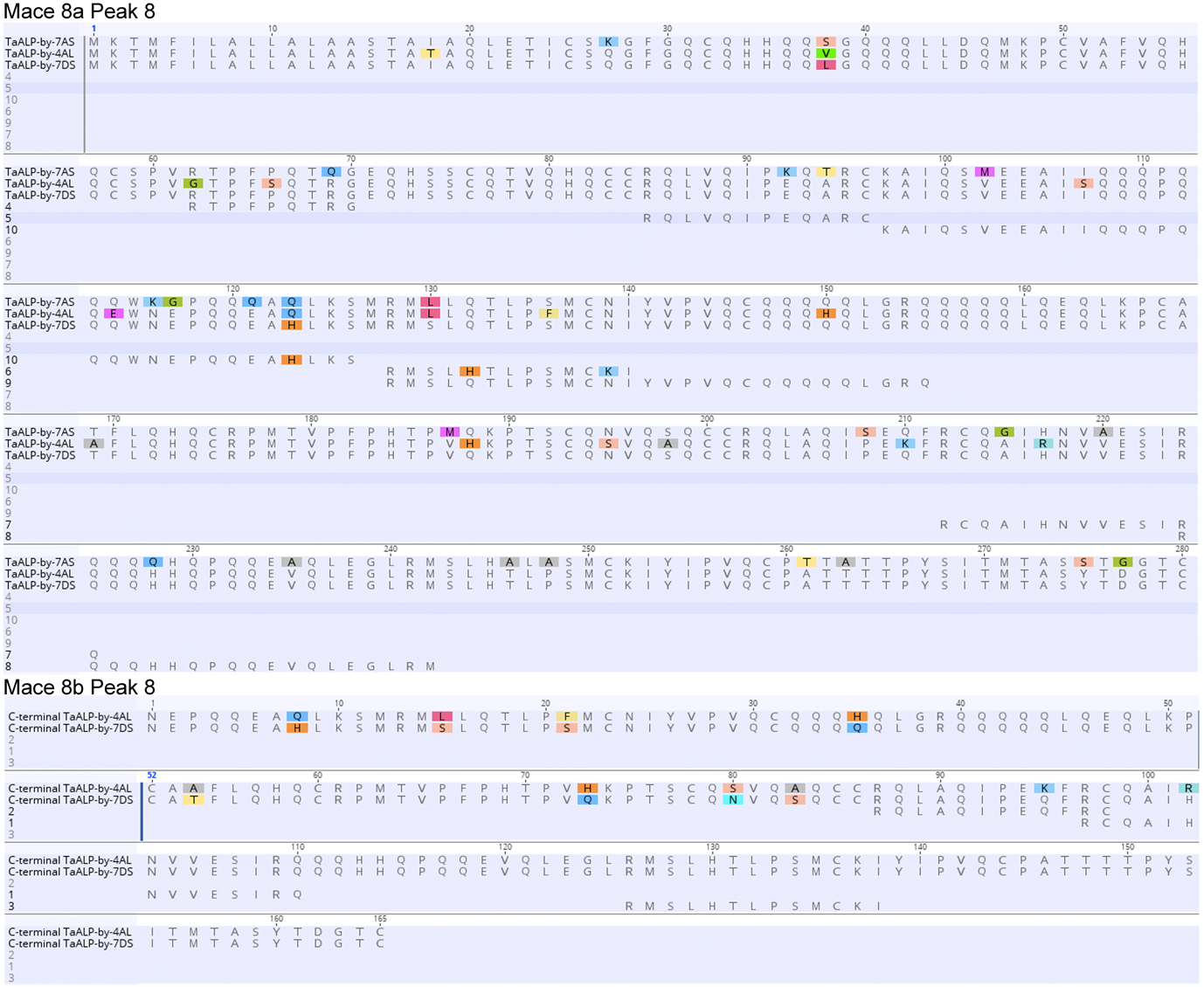

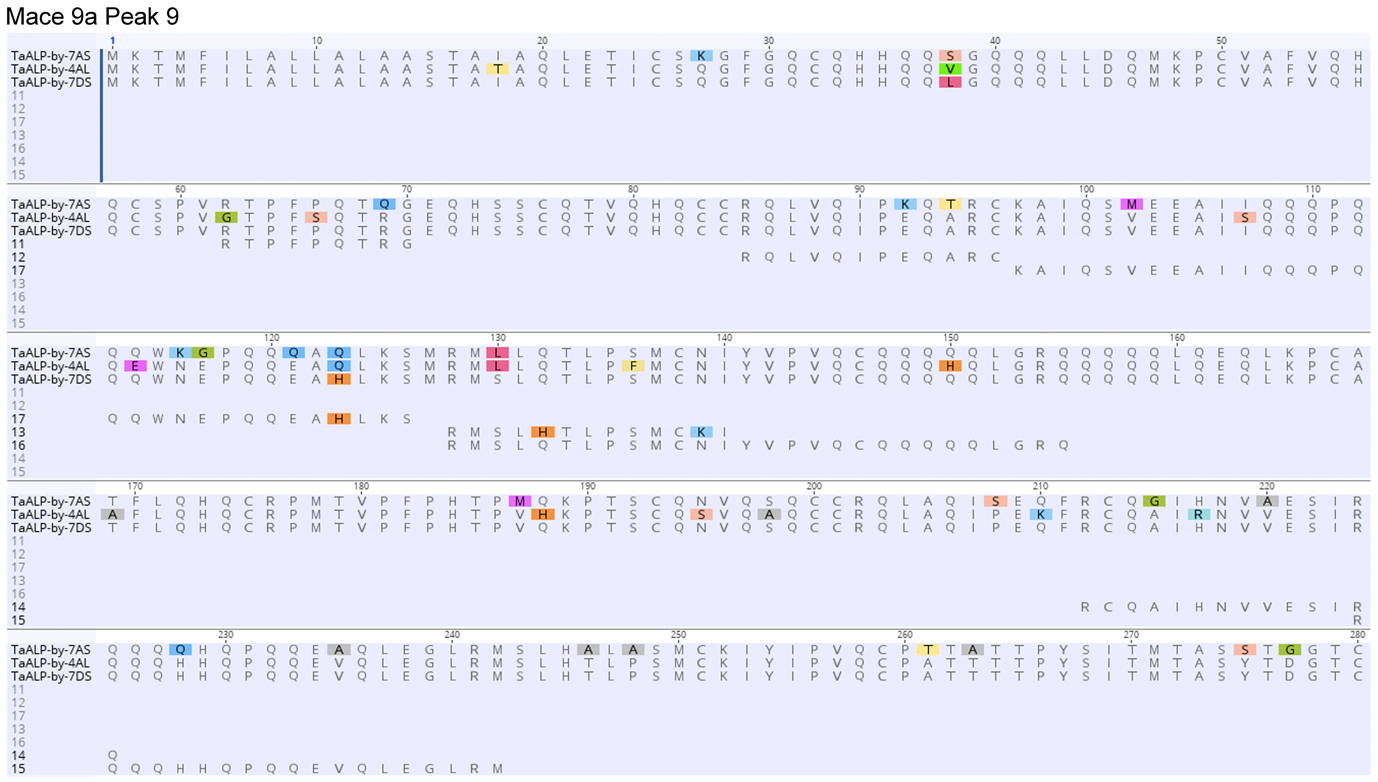

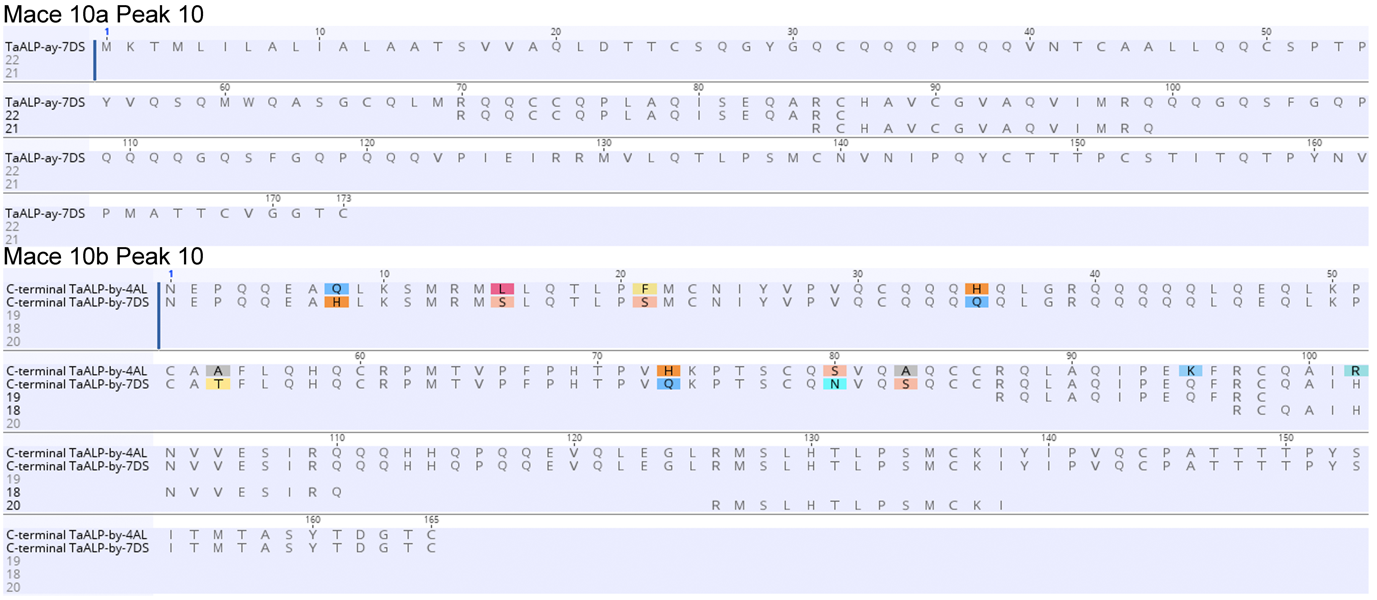

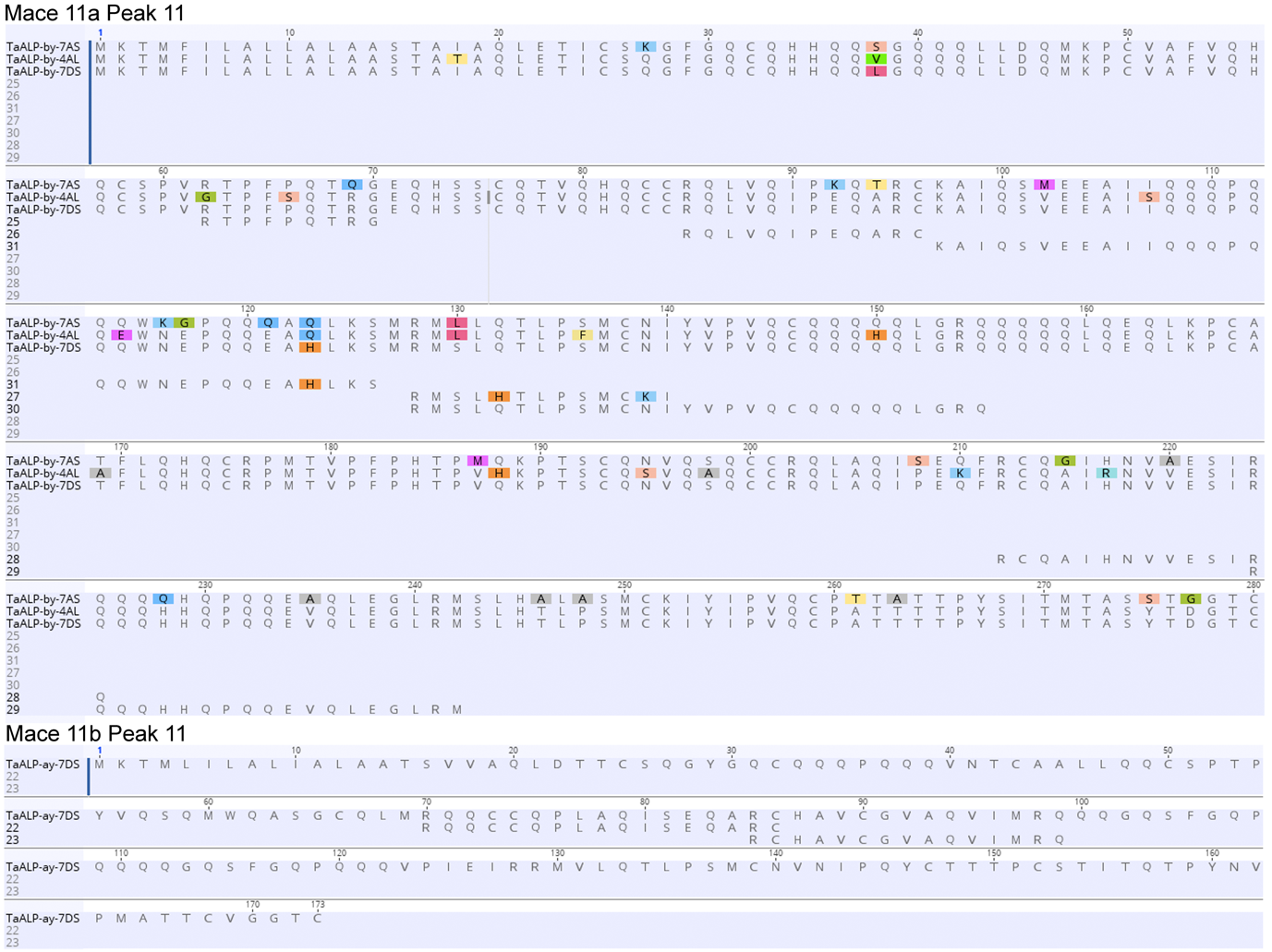

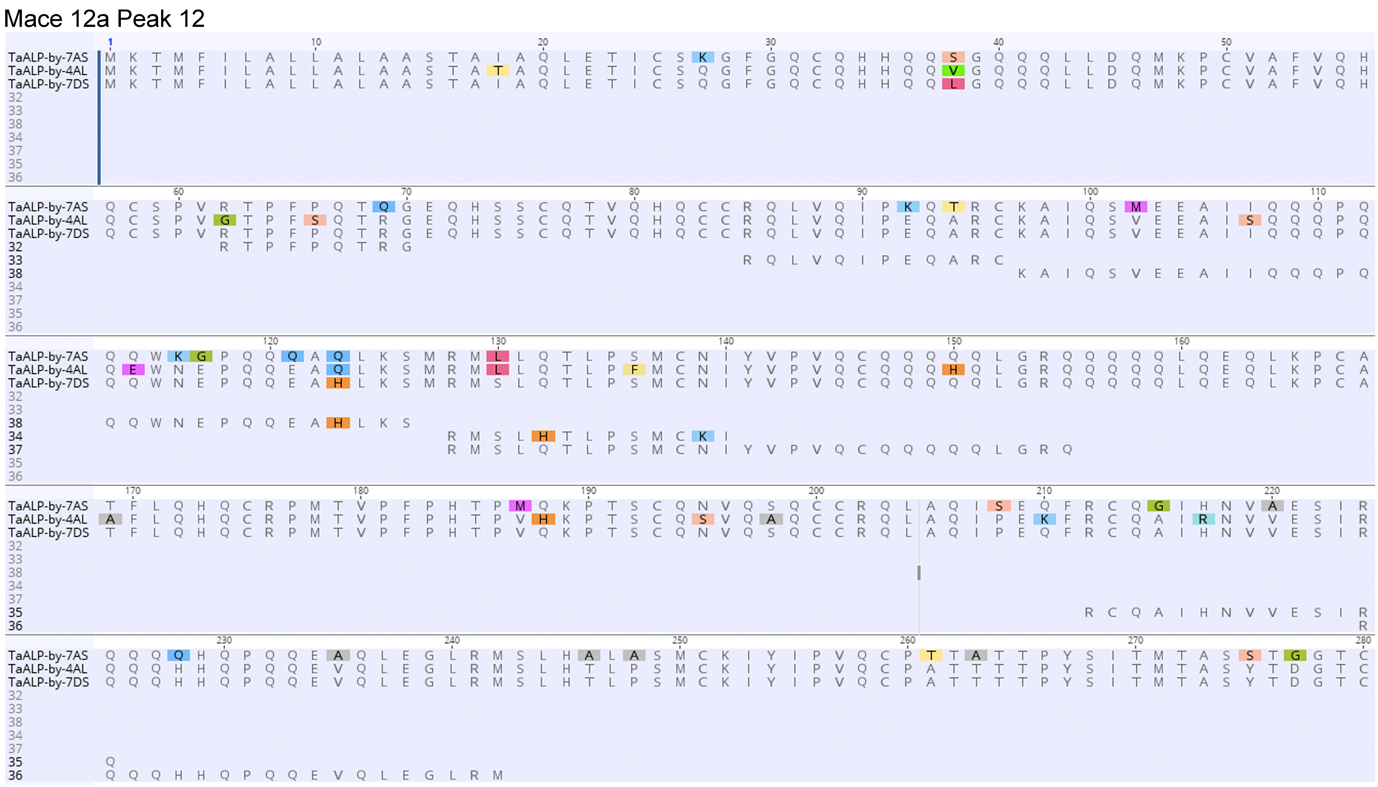

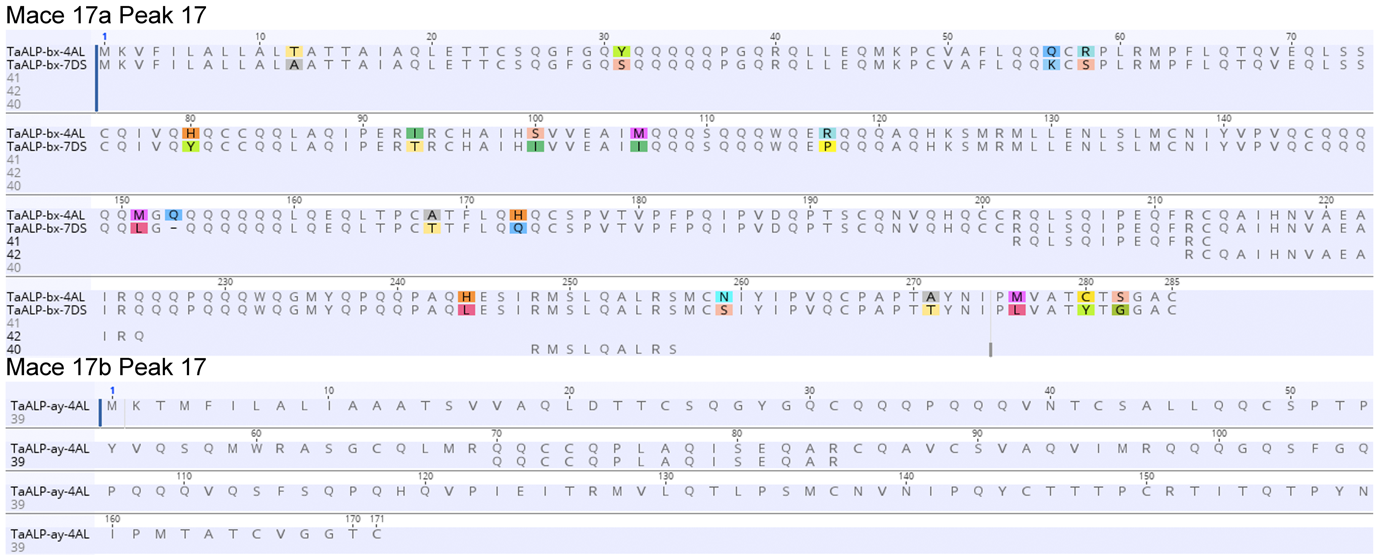

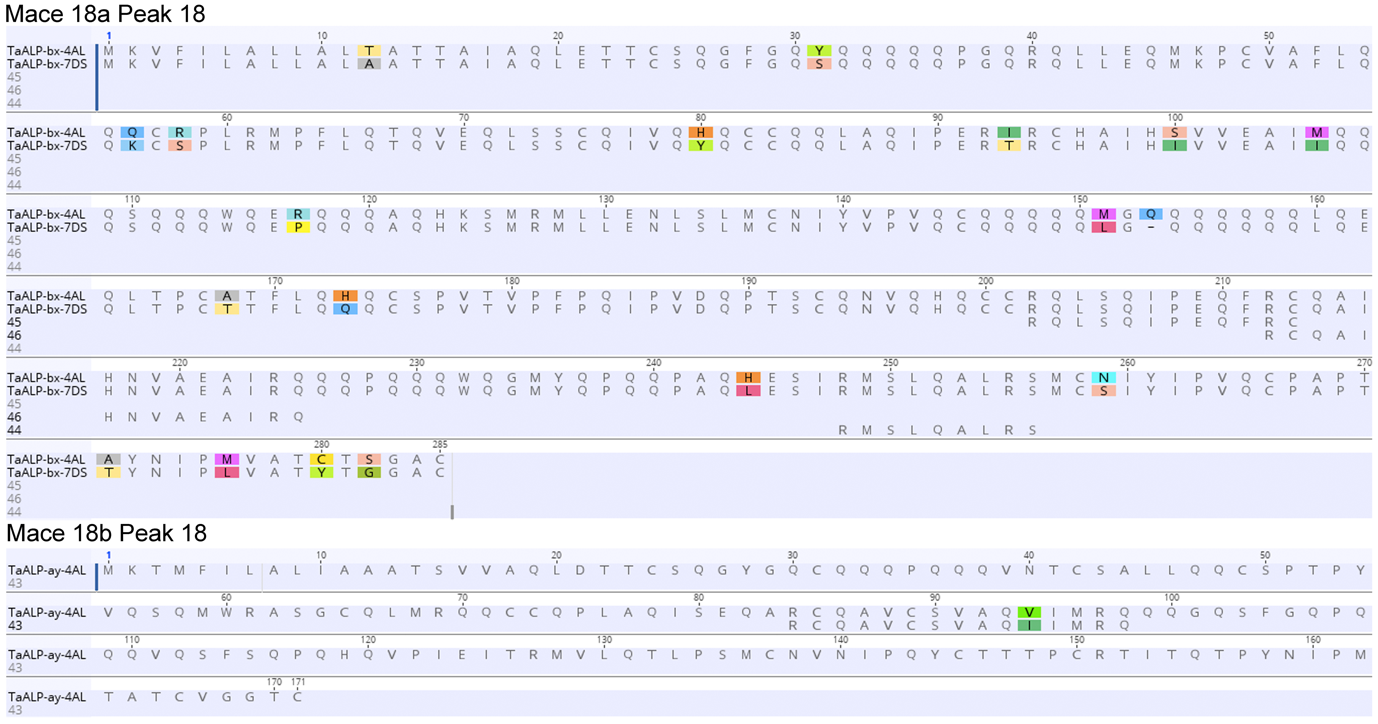

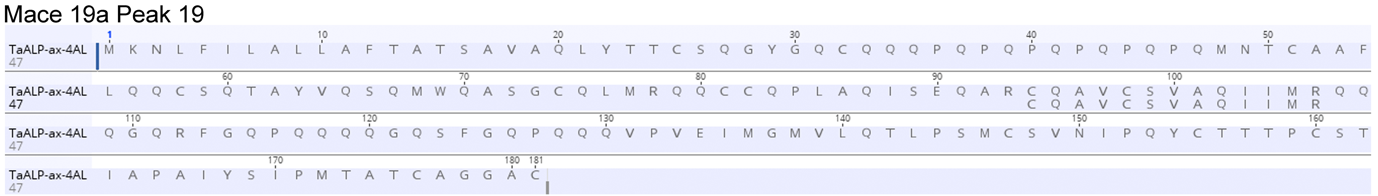

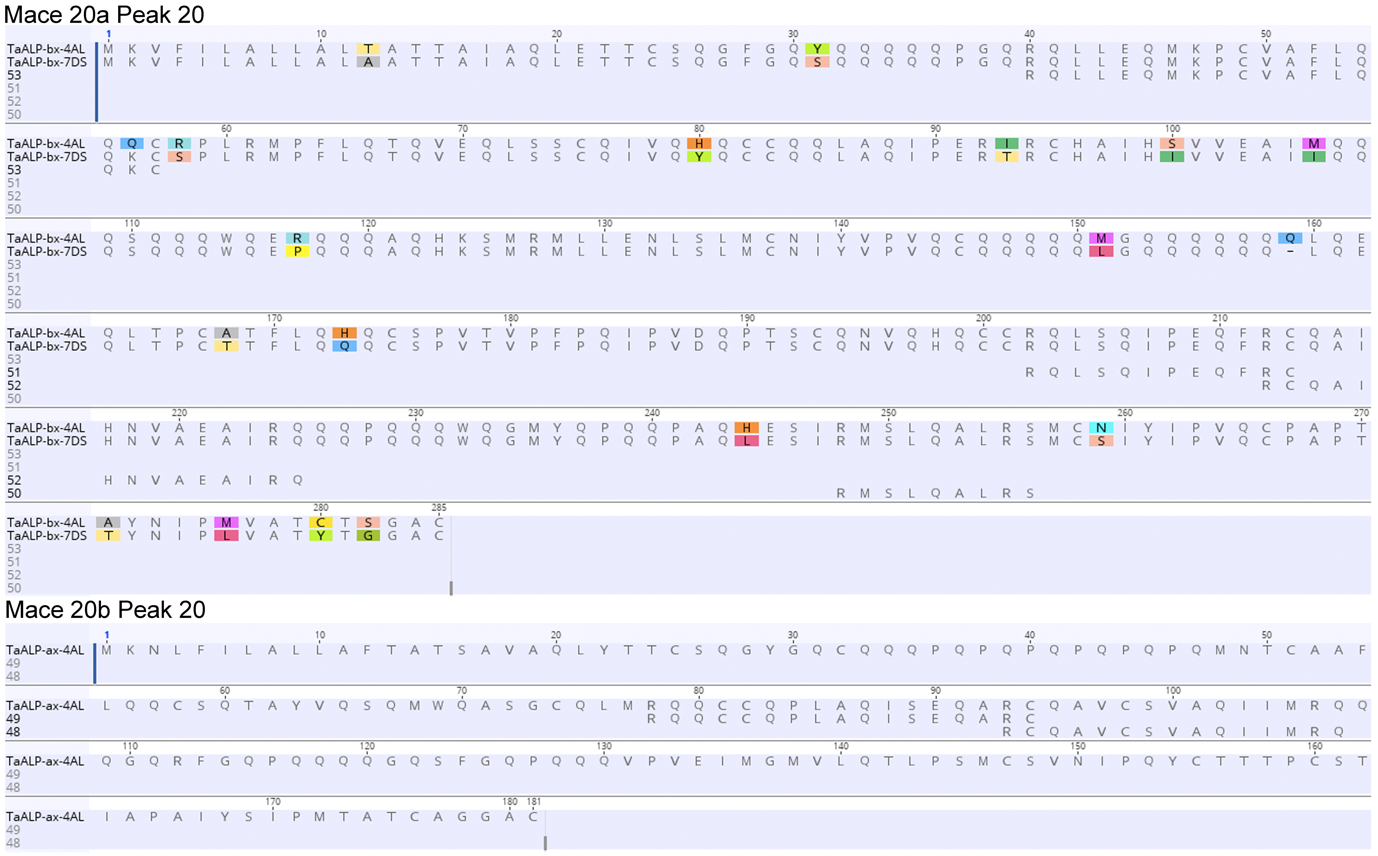

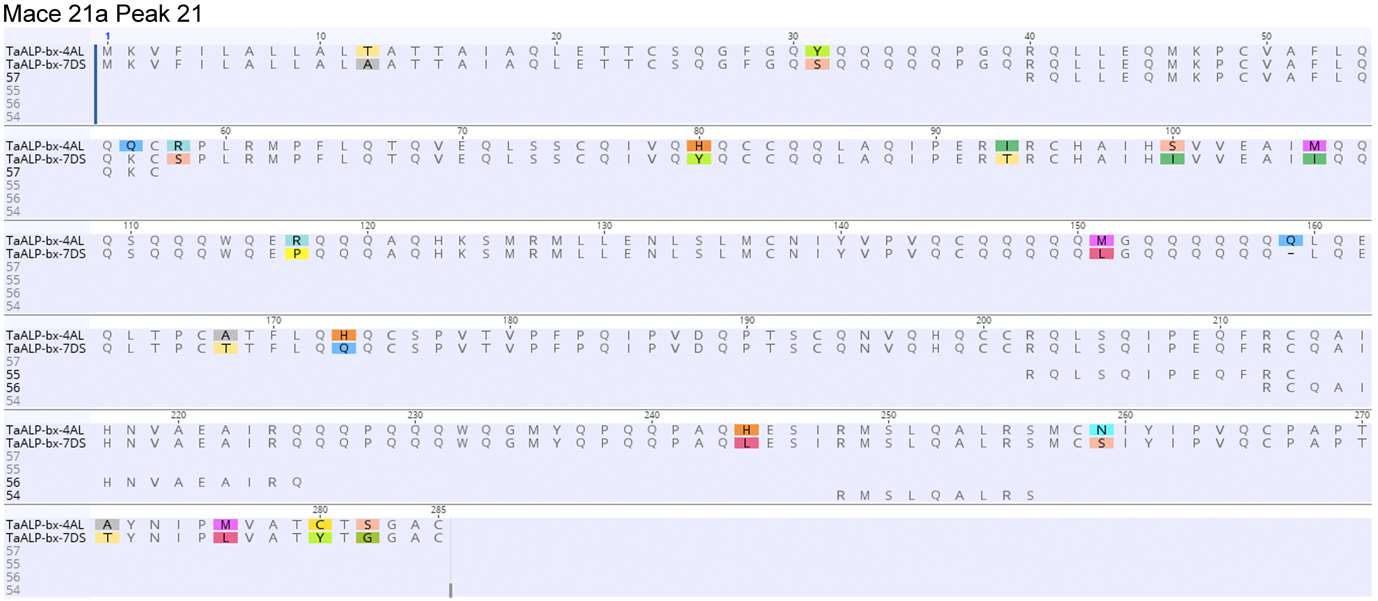

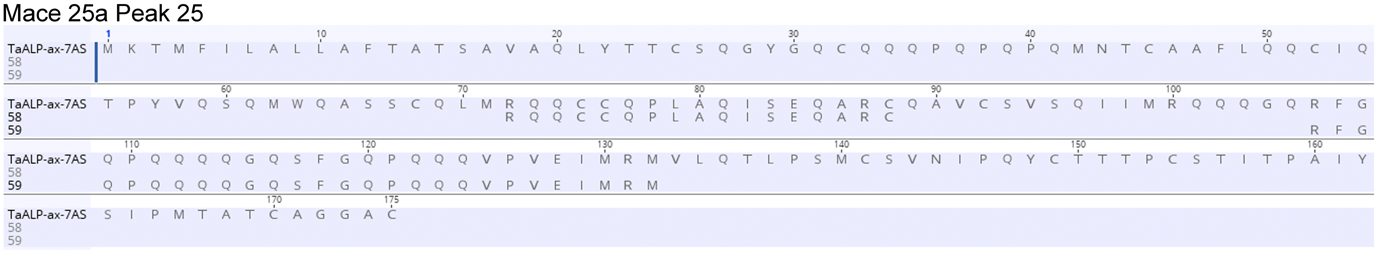

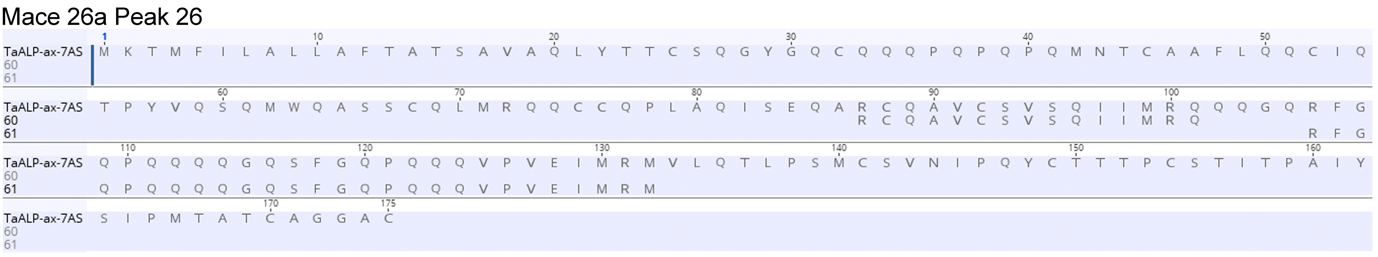

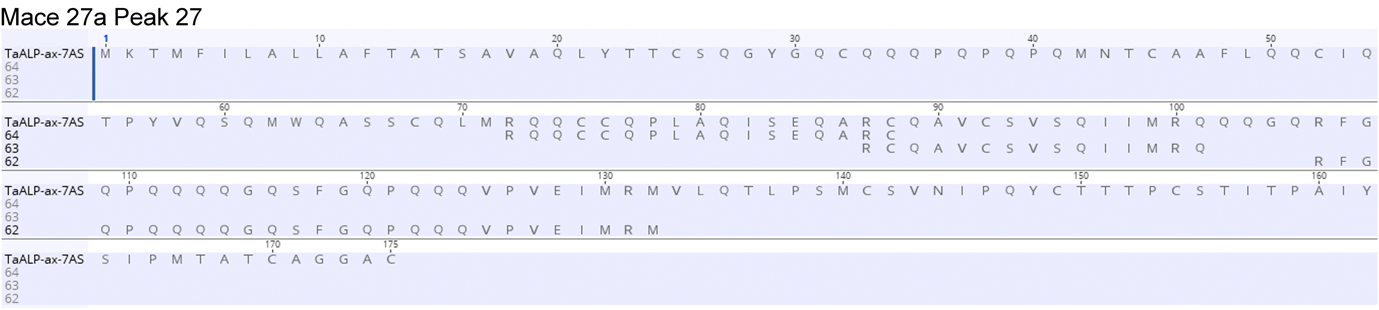

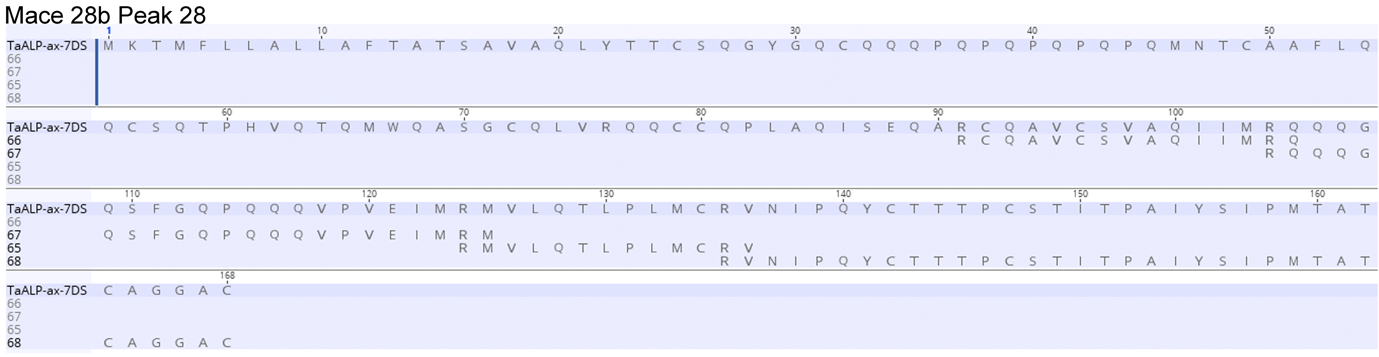

Supplement: Supplementary file 3 — Additional file 3. ALP proteins identification from wheat cv. Mace. [file 12870_2020_2259_MOESM3_ESM.docx]

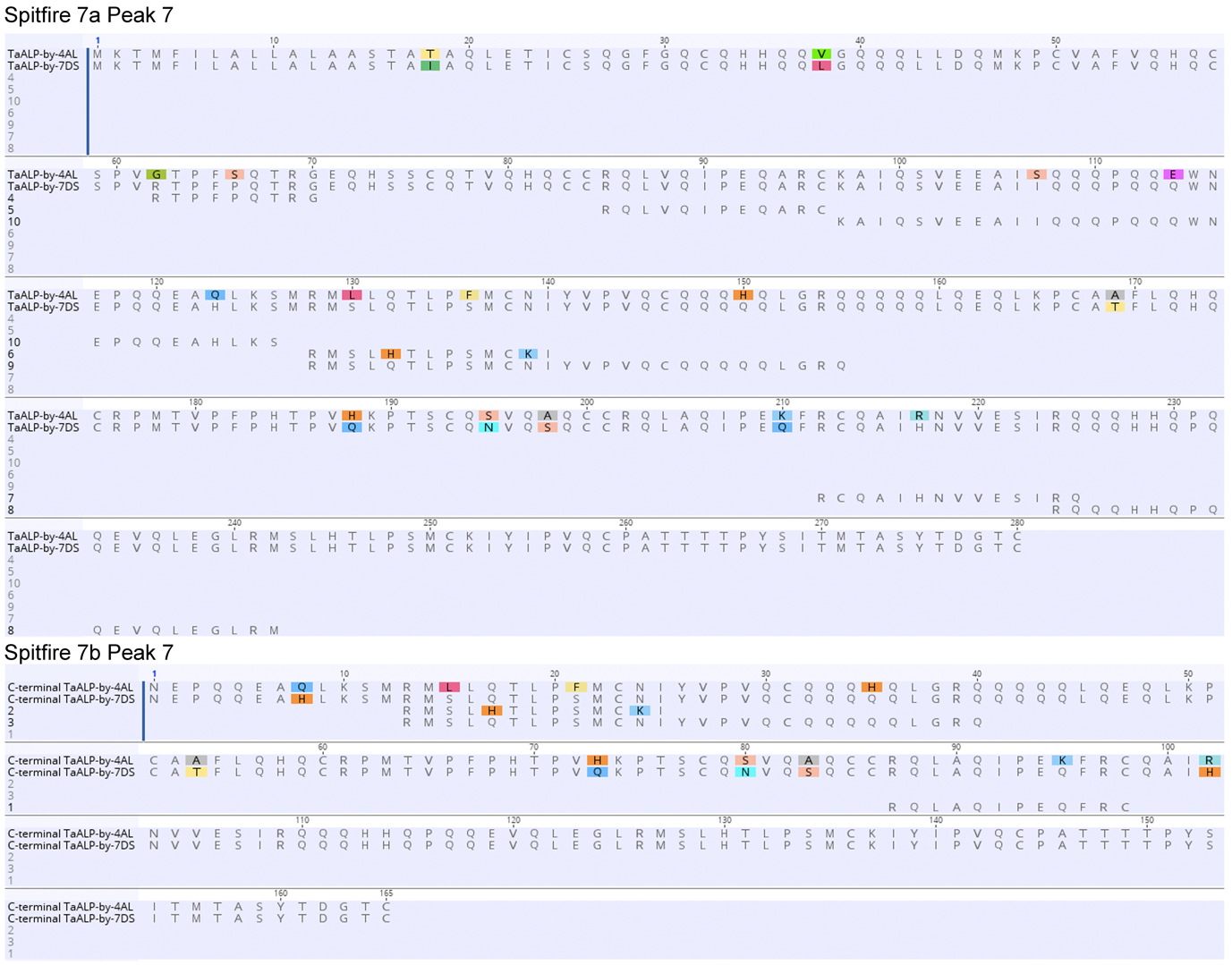

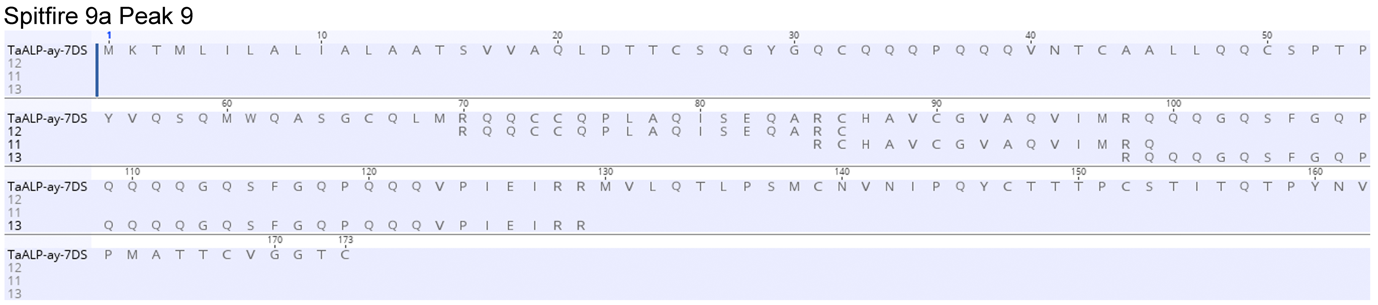

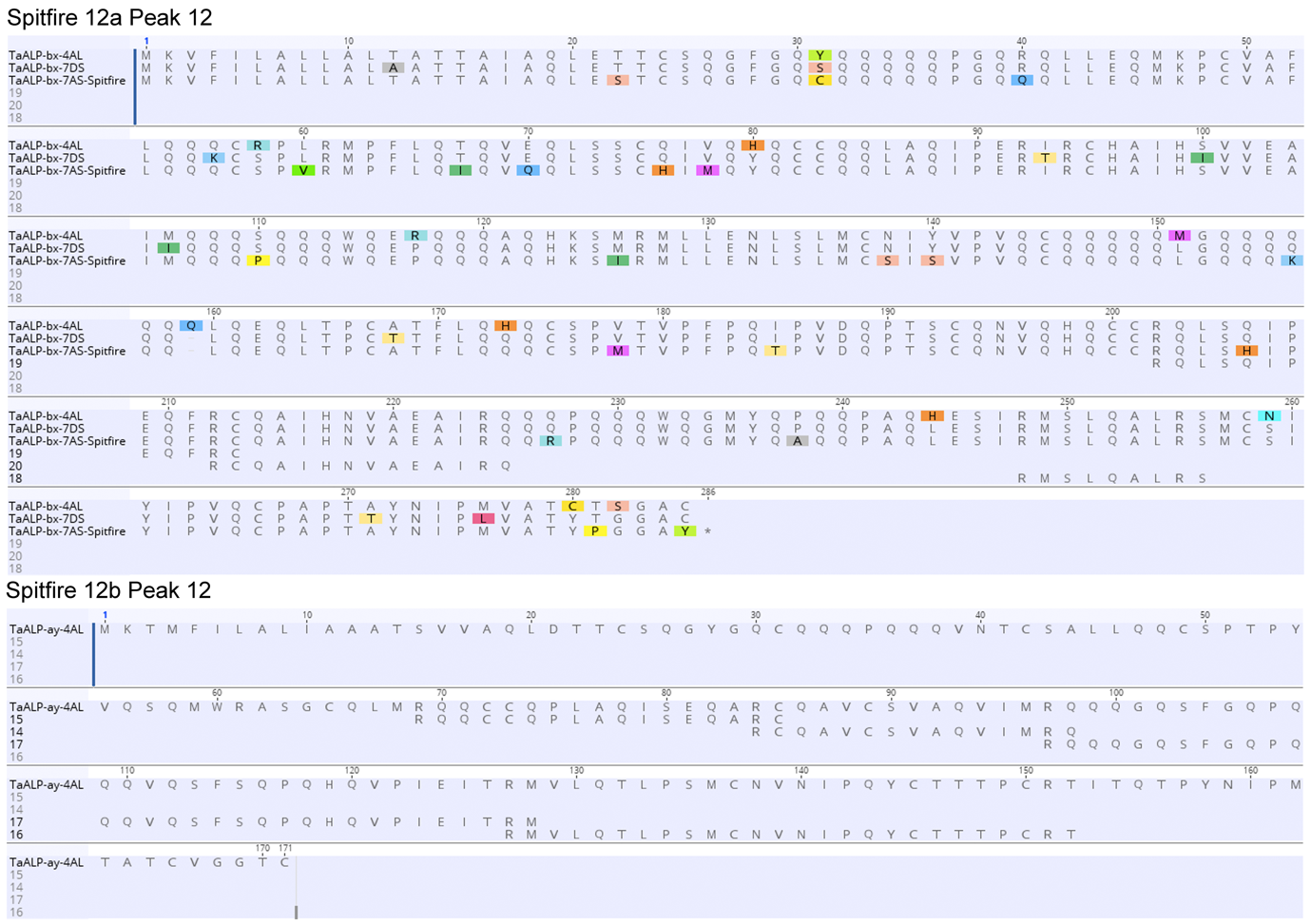

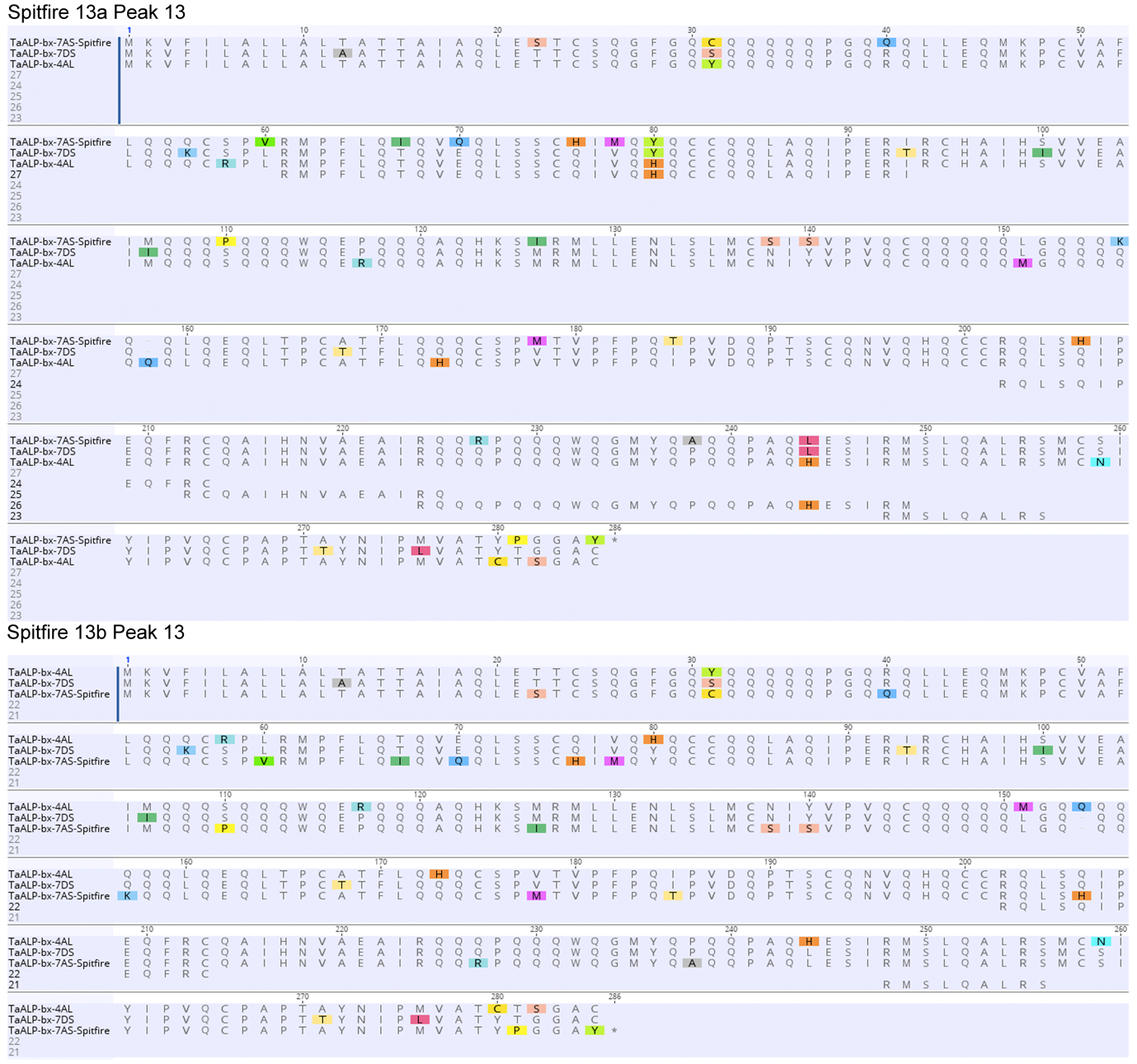

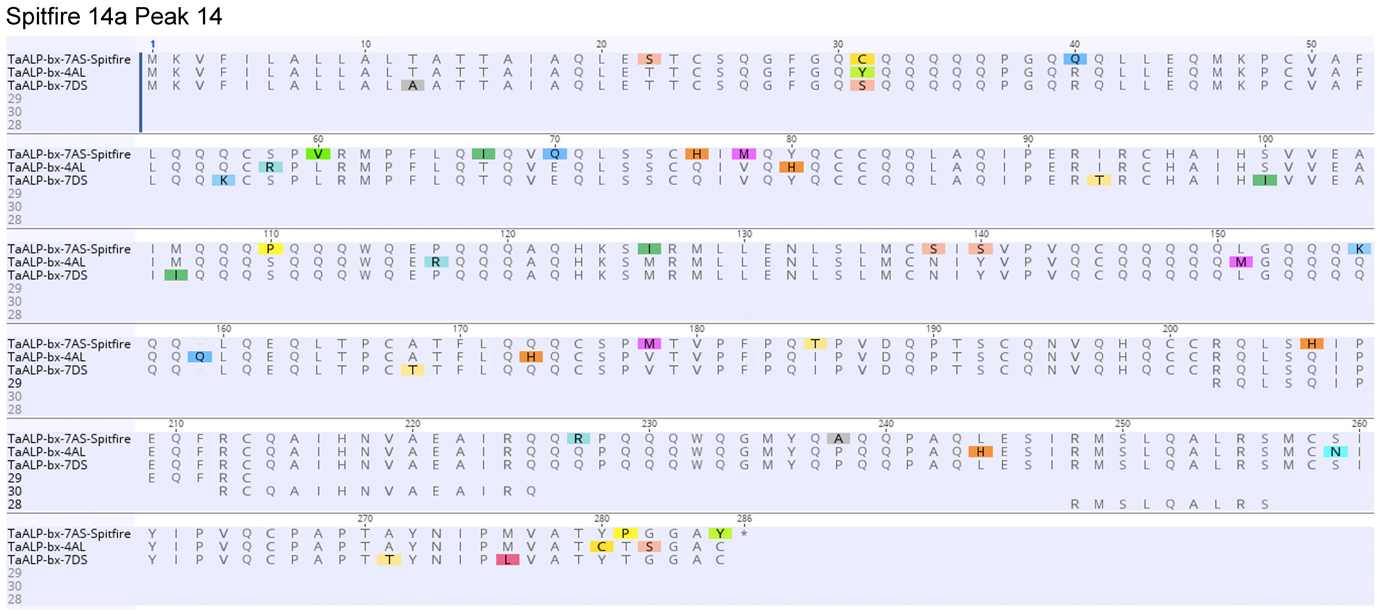

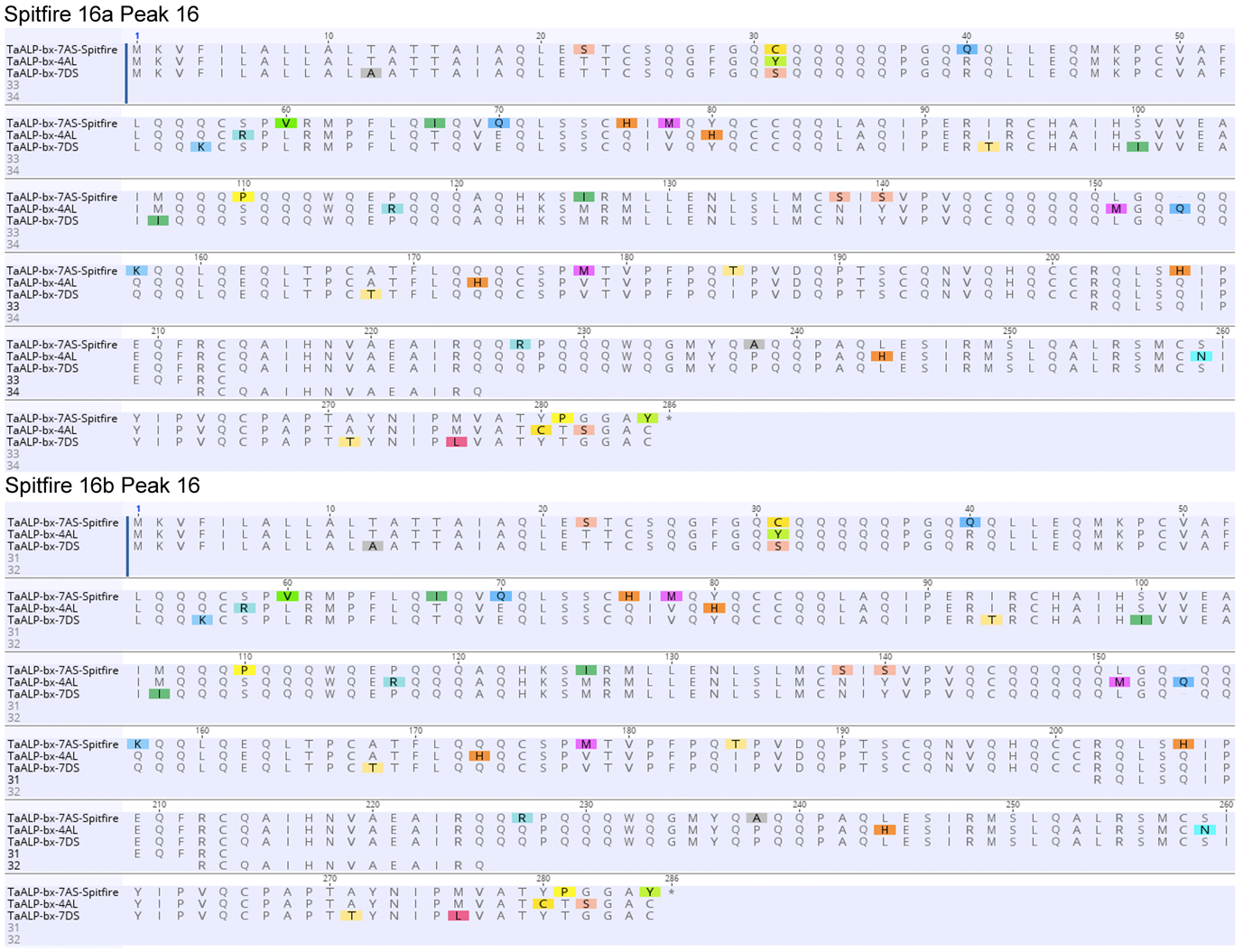

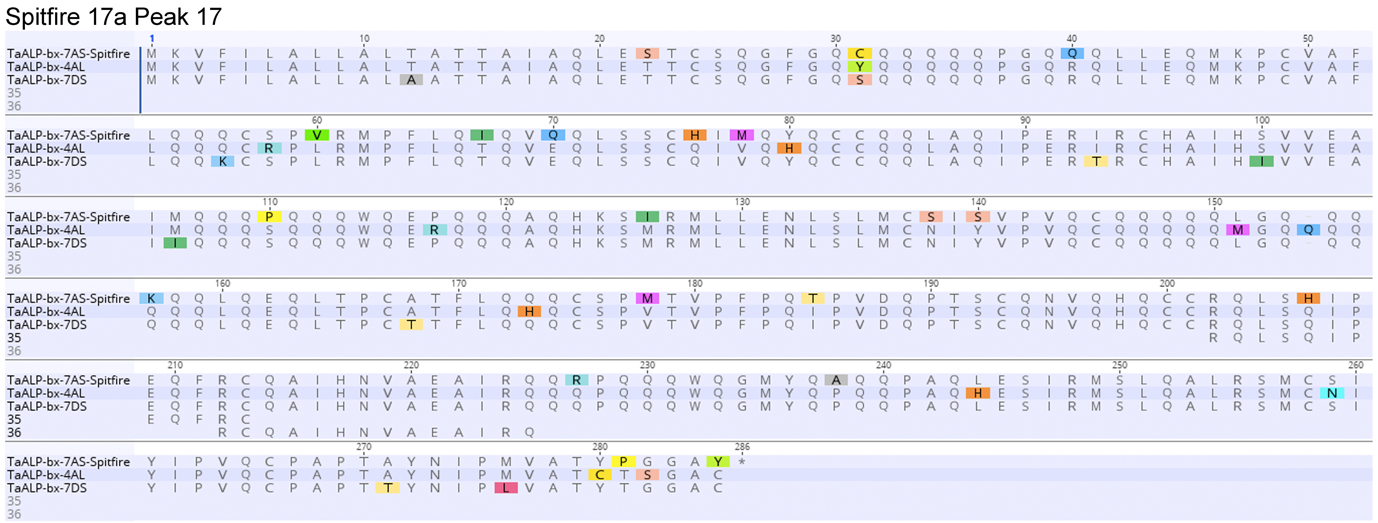

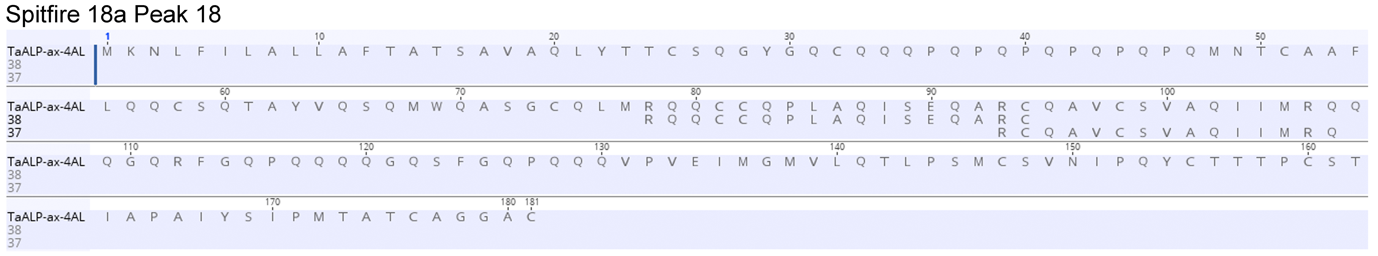

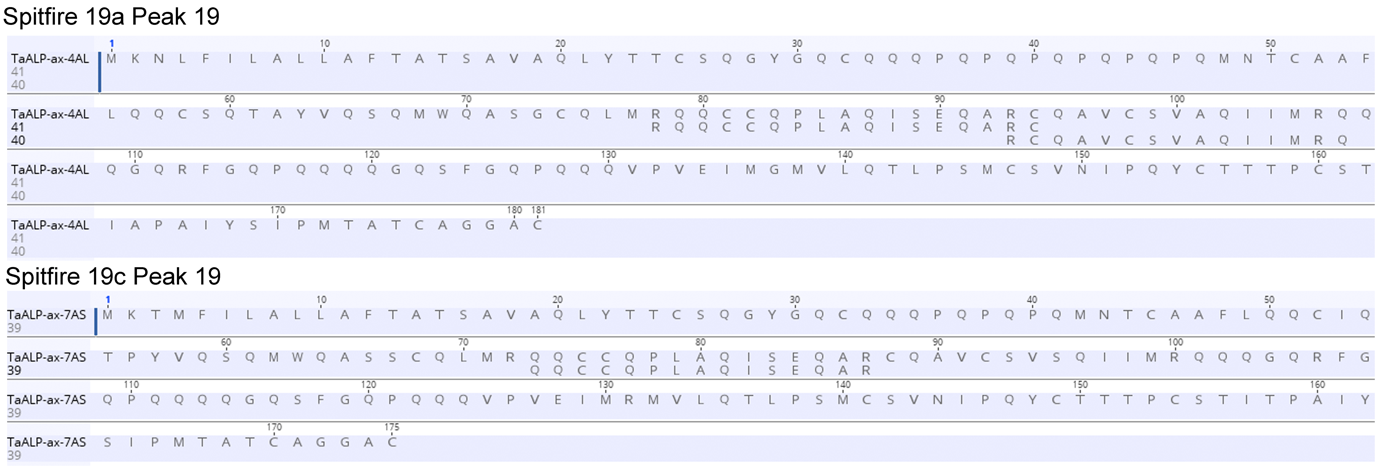

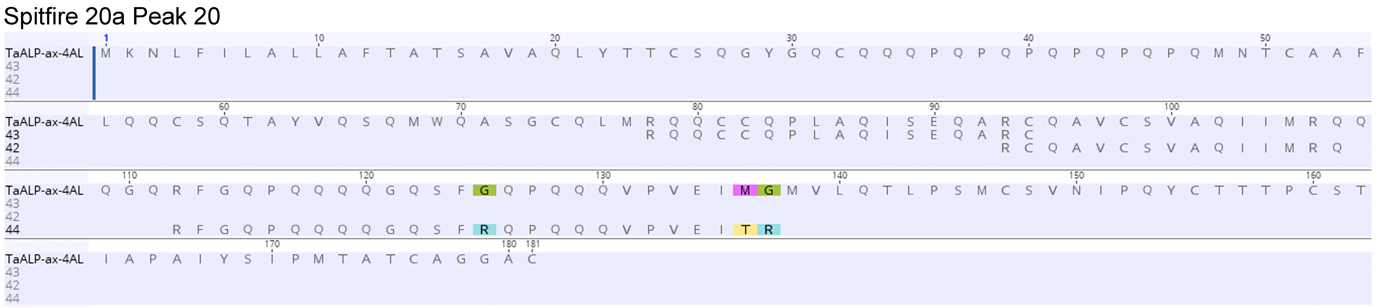

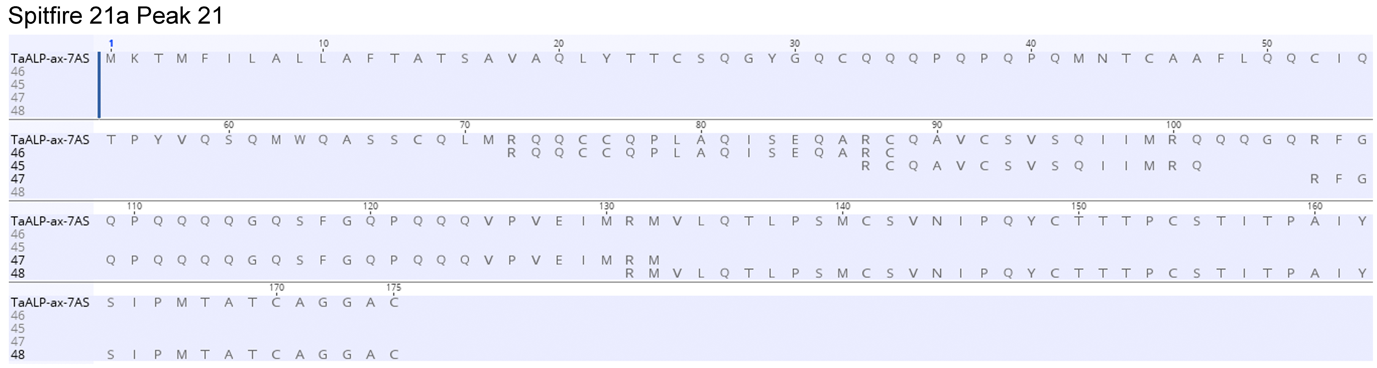

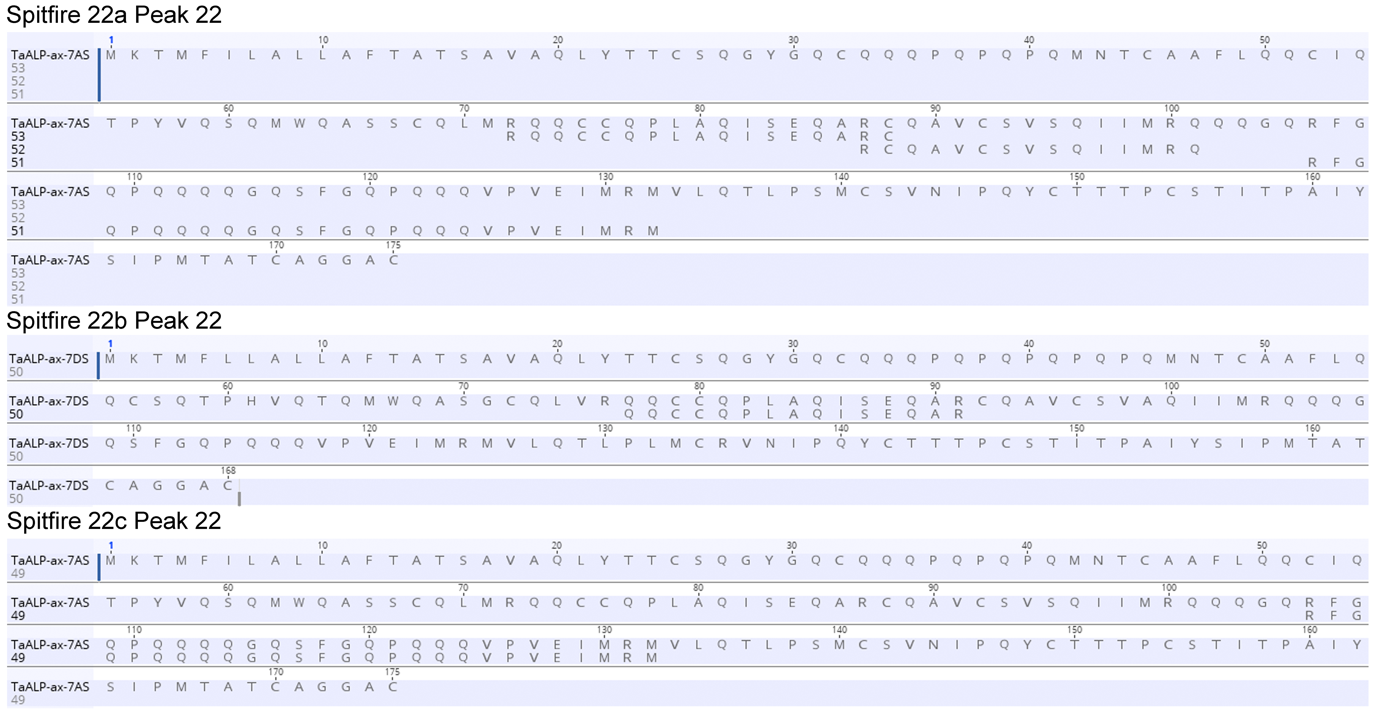

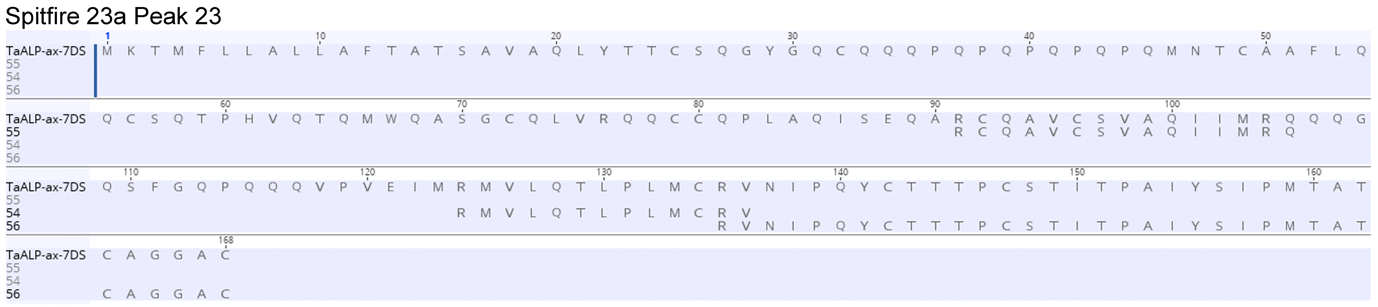

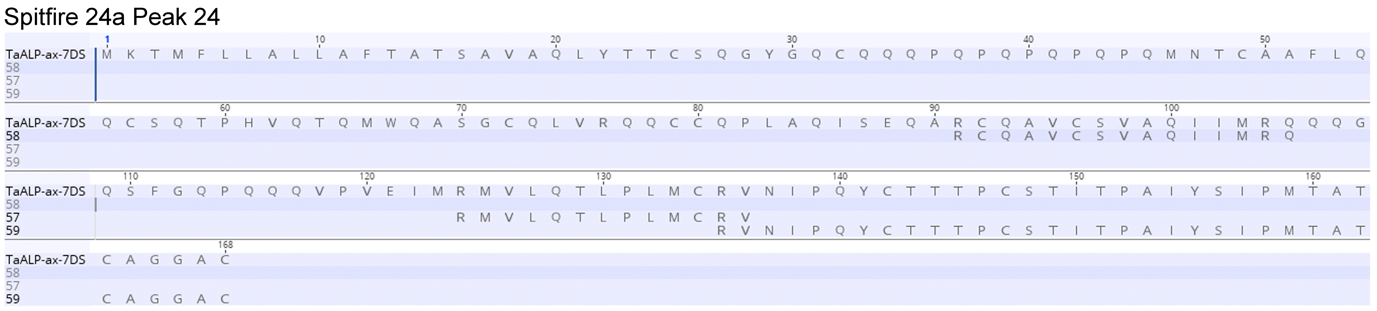

Supplement: Supplementary file 4 — Additional file 4. ALP proteins identification from wheat cv Spitfire. [file 12870_2020_2259_MOESM4_ESM.docx]
