# Supplementary material for: Characterising avenin-like proteins (ALPs) from albumin/globulin fraction of wheat grains by RP-HPLC, SDS-PAGE, and MS/MS peptides sequencing
Source: BMC Plant Biol. 2020 Jan 29;20:45. doi: 10.1186/s12870-020-2259-z (PMC6988229; doi:10.1186/s12870-020-2259-z)
Supplement: Supplementary file 5 — Additional file 5. Original SDS-PAGE gels for Figs. 3 and 5. [file 12870_2020_2259_MOESM5_ESM.docx]

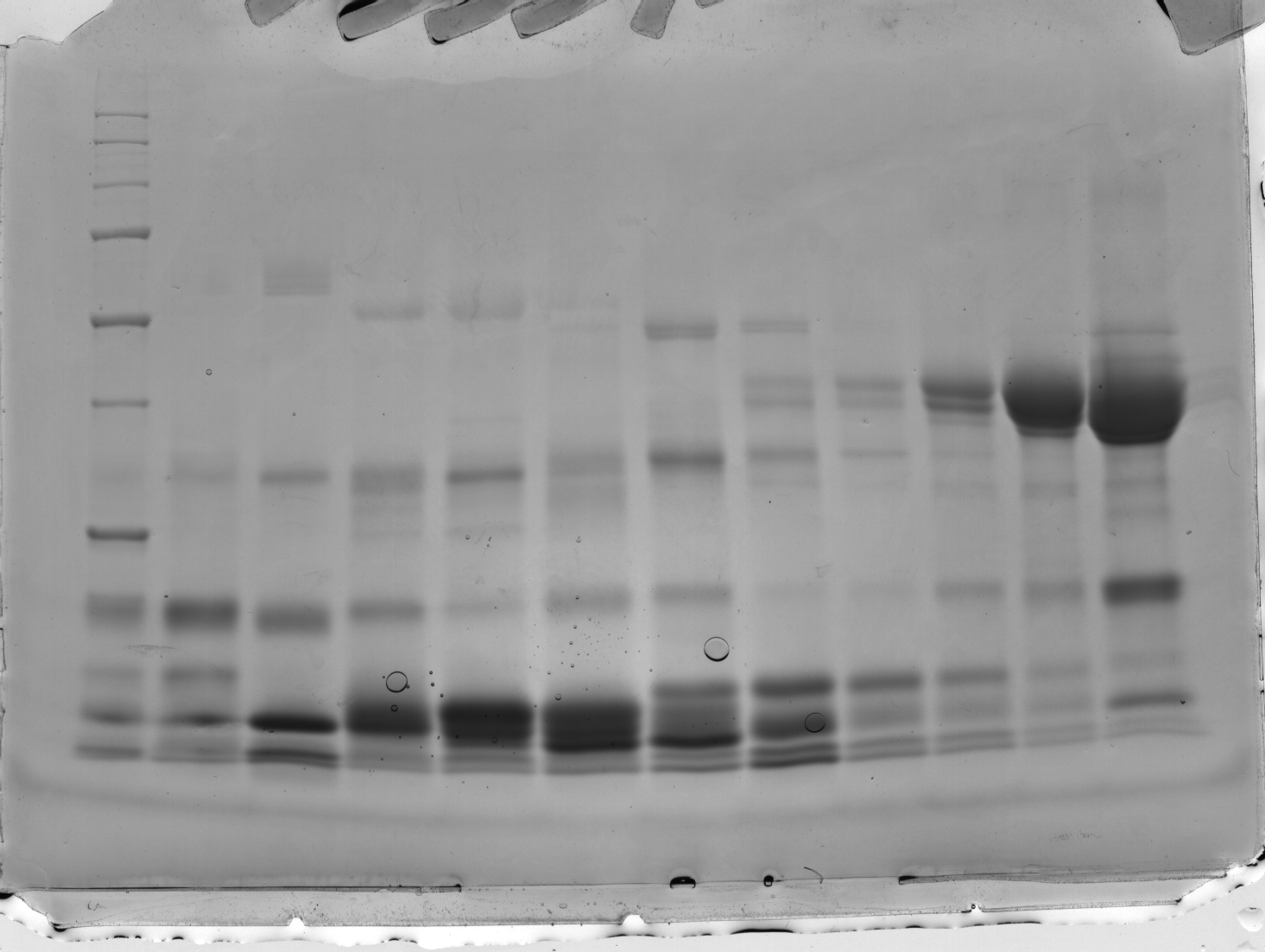


**Figure S1** The original SDS-PAGE gel presented in Figure 3B.


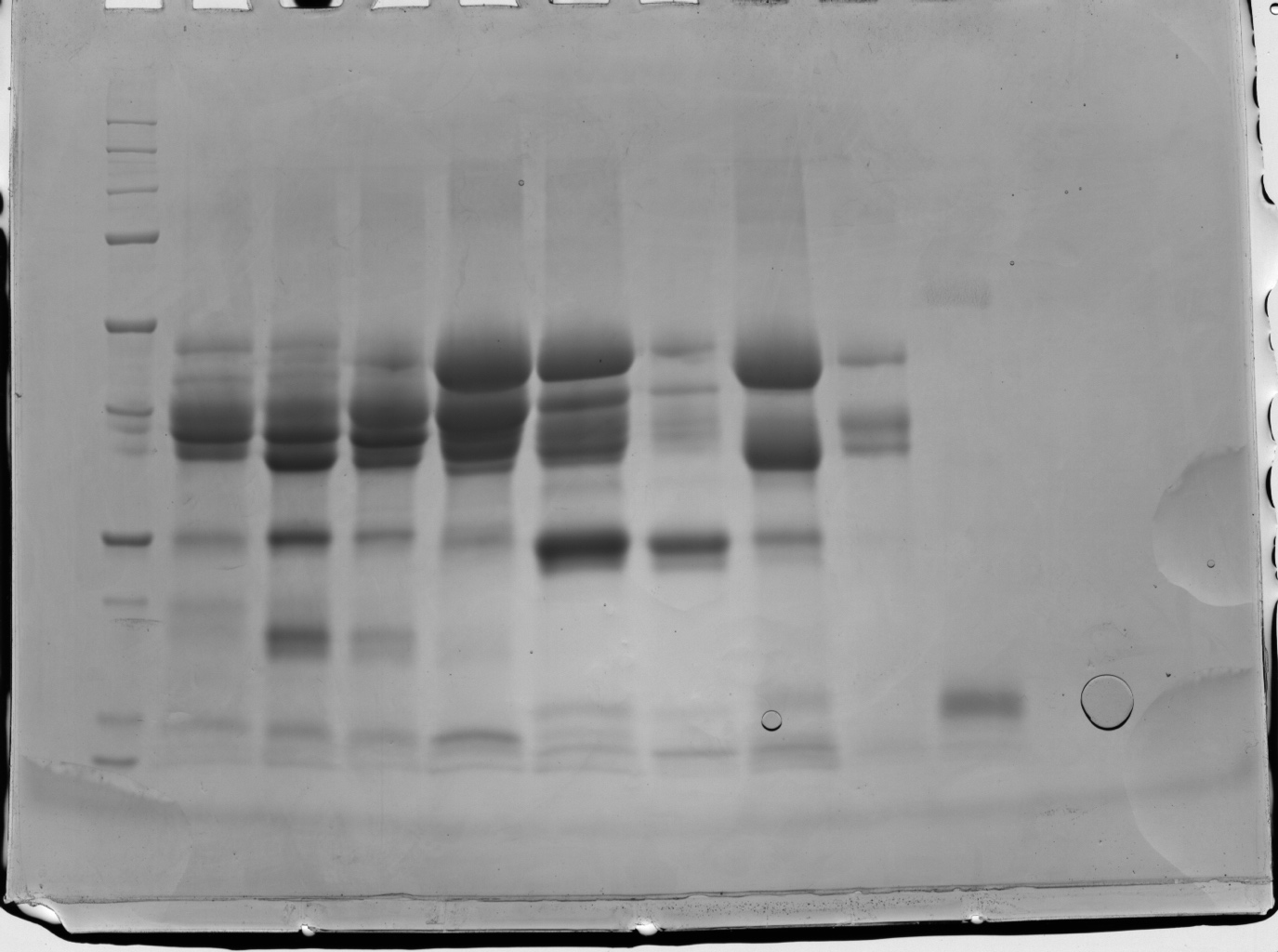


**Figure S2** The original SDS-PAGE gel presented in Figure 3C.


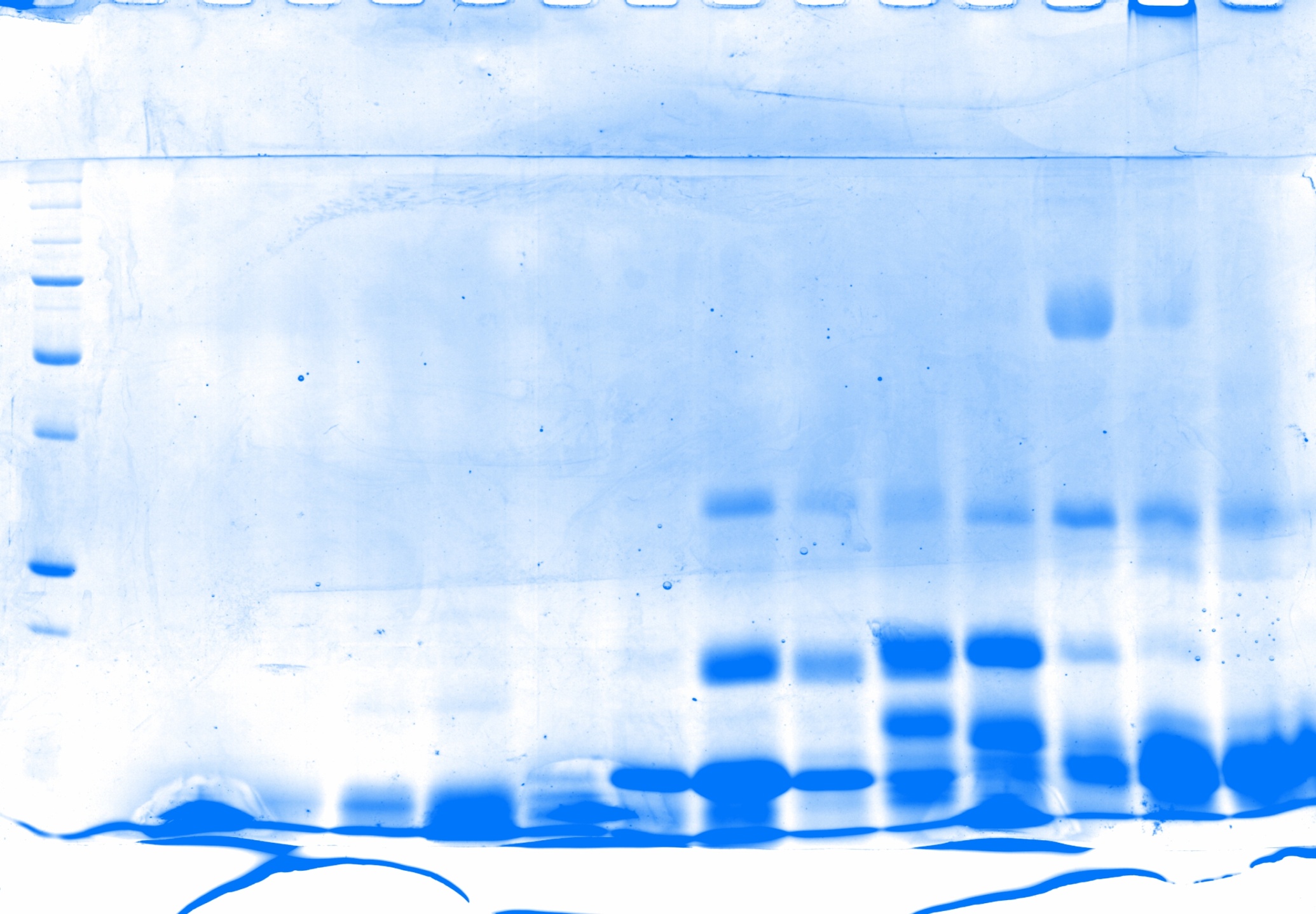


**Figure S3** The original SDS-PAGE gel presented in Figure 5B for peaks 1 to 14.


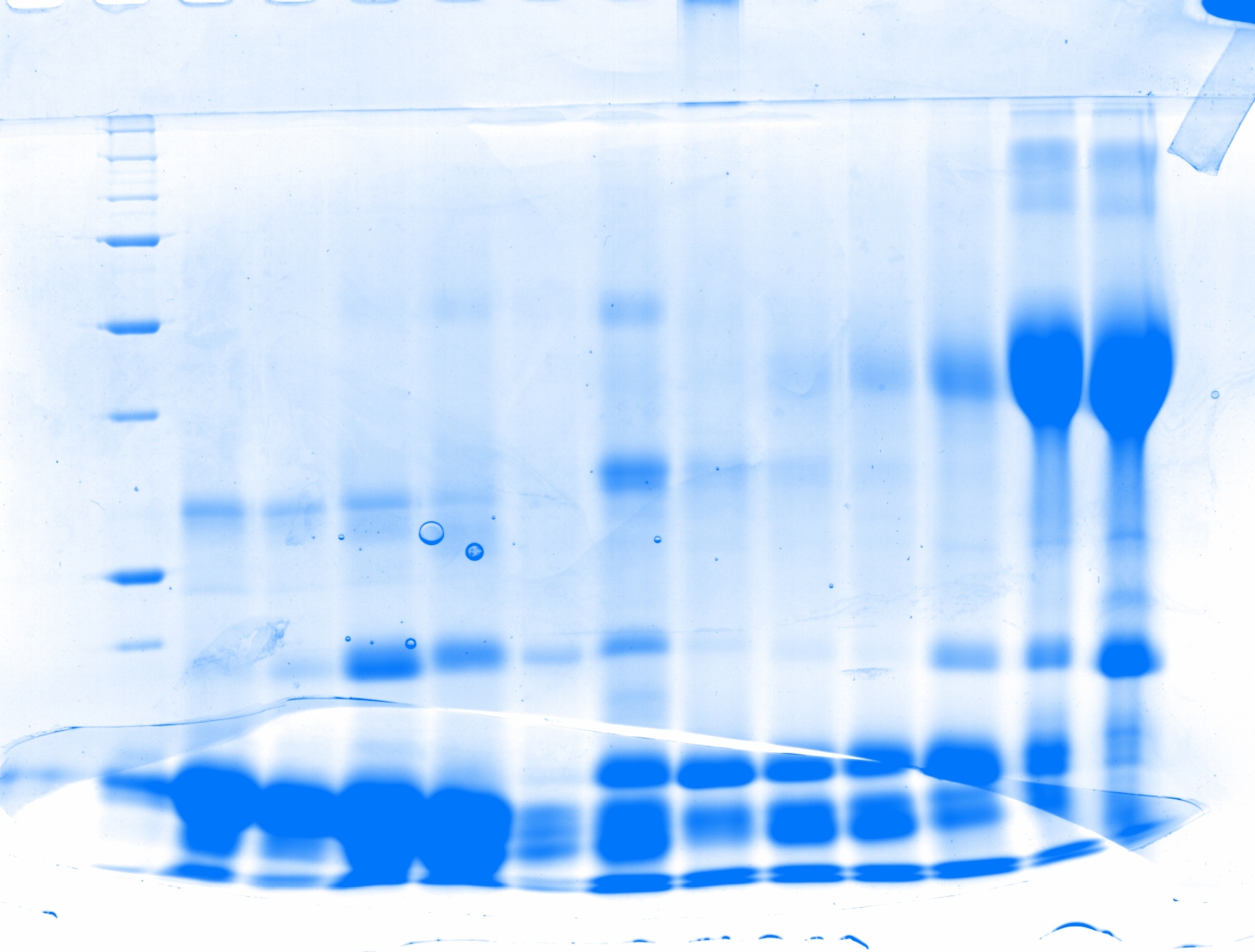


**Figure S4** The original SDS-PAGE gel presented in Figure 5B for peaks 15 to 26.


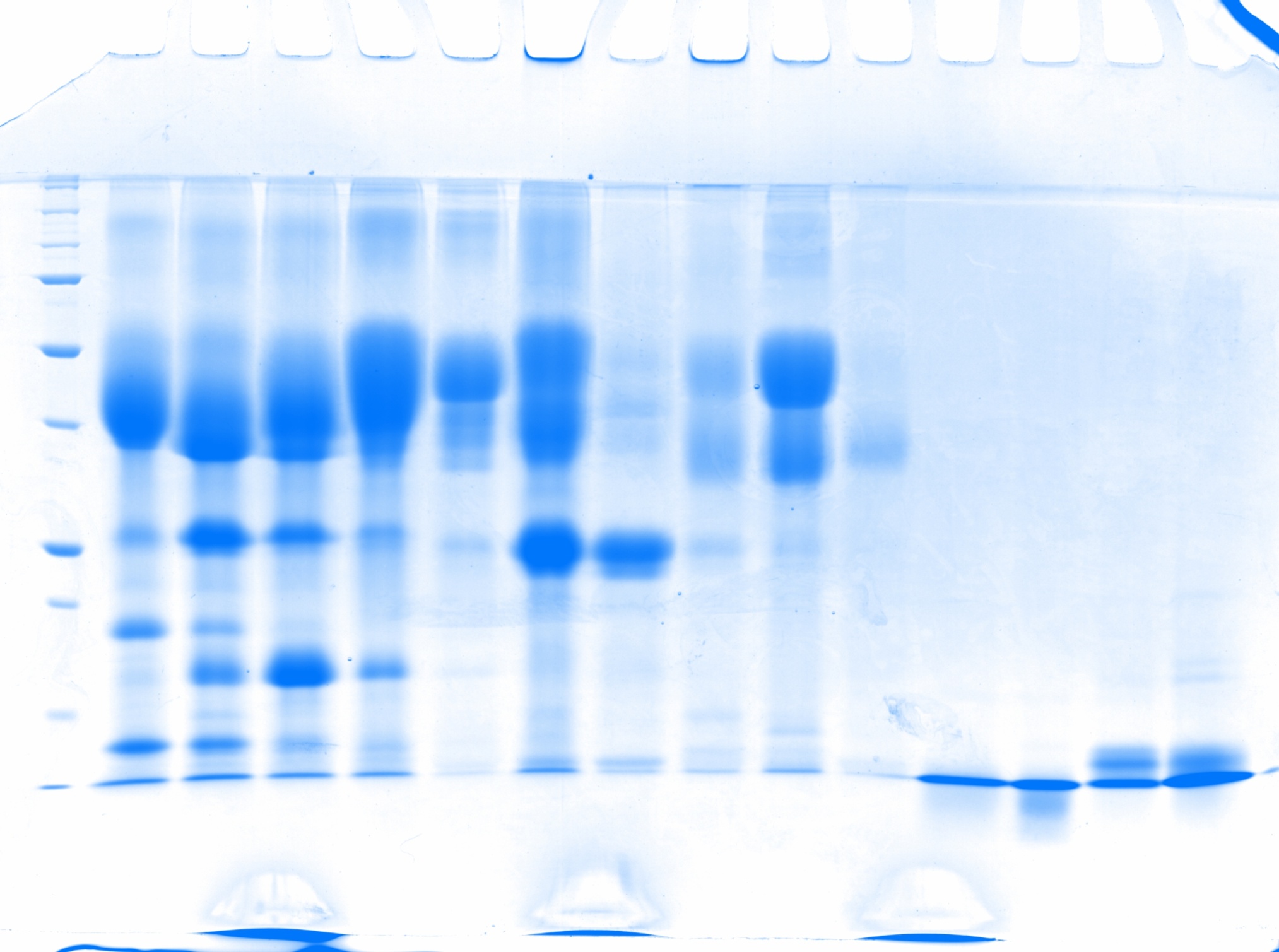


**Figure S5** The original SDS-PAGE gel presented in Figure 5B for peaks 27 to 36, and the original SDS-PAGE gel presented in Figure 5D for peaks 1 to 4.


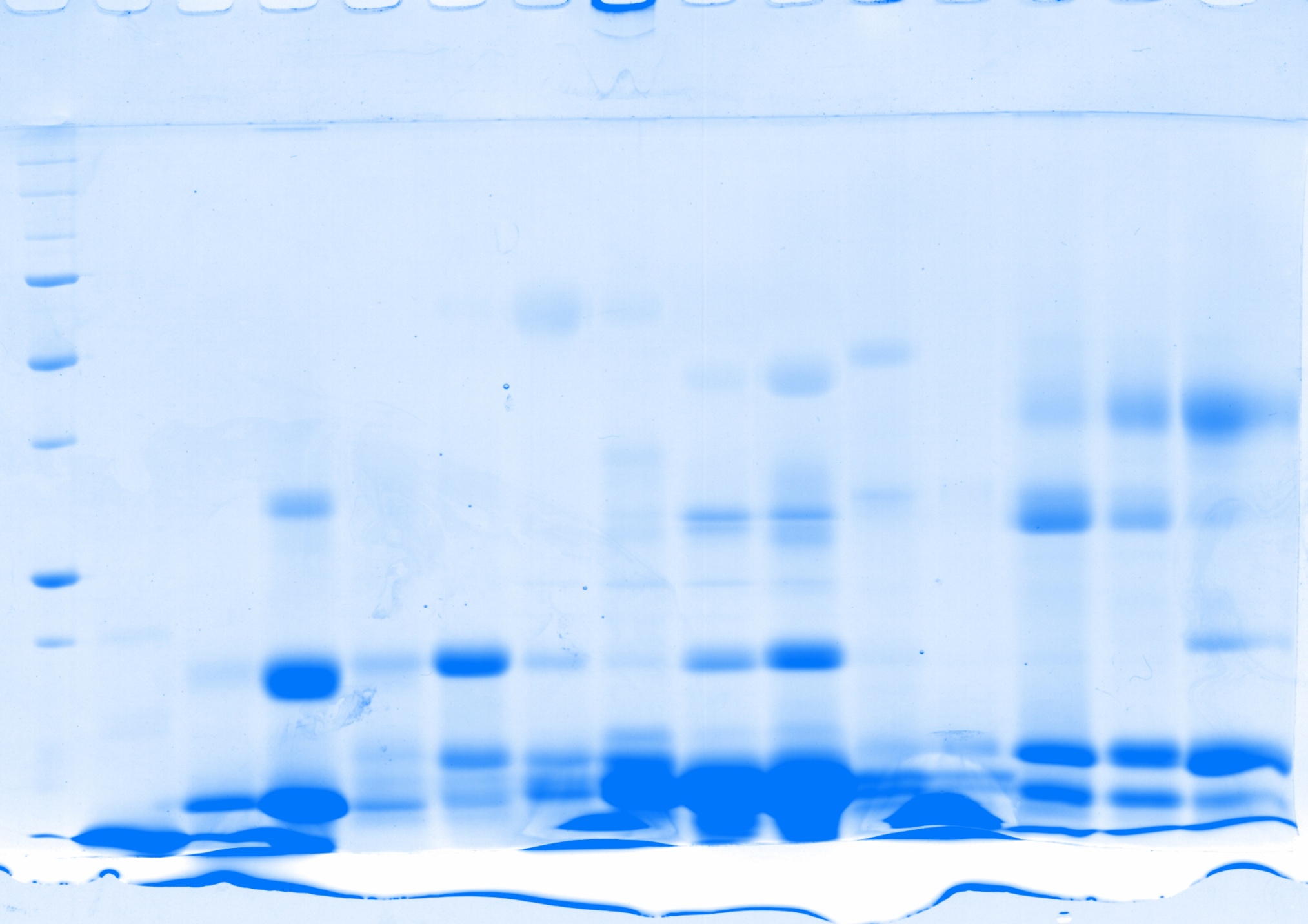


**Figure S6** The original SDS-PAGE gel presented in Figure 5D for peaks 5 to 18.


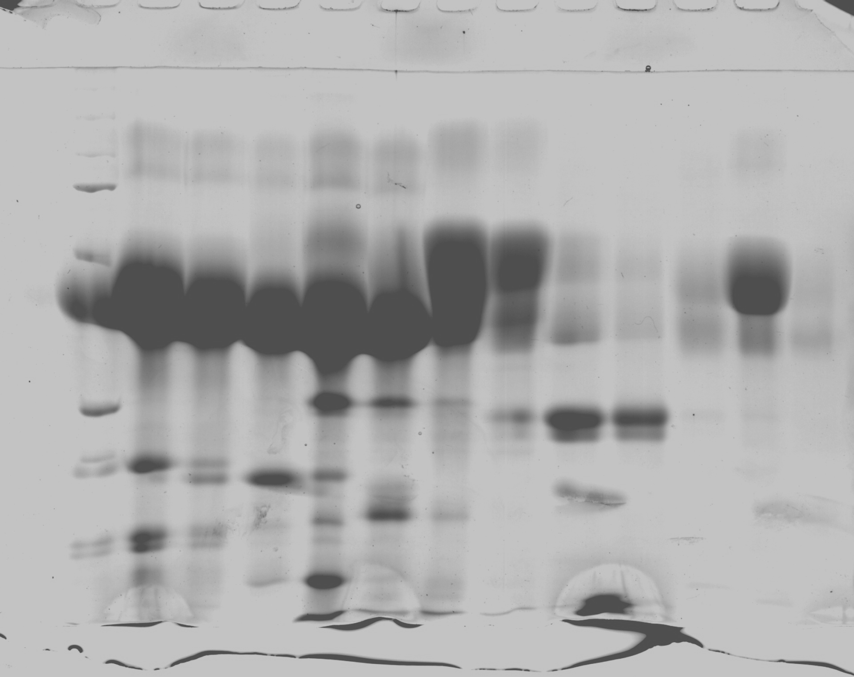


**Figure S7** The original SDS-PAGE gel presented in Figure 5B for peaks 19 to 30.
